# AMPK promotes Arf6 activation in a kinase-independent manner upon glucose starvation

**DOI:** 10.1242/jcs.259609

**Published:** 2022-09-14

**Authors:** Kuan-Jung Chen, Jia-Wei Hsu, Fang-Jen S. Lee

**Affiliations:** ^1^ Institute of Molecular Medicine, College of Medicine, National Taiwan University, Taipei 100, Taiwan; ^2^ Center of Precision Medicine, College of Medicine, National Taiwan University, Taipei 100, Taiwan; ^3^ Institute of Biochemical Sciences, College of Life Science, National Taiwan University, Taipei 106, Taiwan; ^4^ Institute of Biological Chemistry, Academia Sinica, Taipei 115, Taiwan; ^5^ Department of Medical Research, National Taiwan University Hospital, Taipei 100, Taiwan

**Keywords:** ADP-ribosylation factor, GTPase, Glucose deprivation, Cell invasion

## Abstract

AMP-activated protein kinase (AMPK) is a crucial cellular nutrient and energy sensor that maintains energy homeostasis. AMPK also governs cancer cell invasion and migration by regulating gene expression and activating multiple cellular signaling pathways. ADP-ribosylation factor 6 (Arf6) can be activated via nucleotide exchange by guanine-nucleotide-exchange factors (GEFs), and its activation also regulates tumor invasion and migration. By studying GEF-mediated Arf6 activation, we have elucidated that AMPK functions as a noncanonical GEF for Arf6 in a kinase-independent manner. Moreover, by examining the physiological role of the AMPK–Arf6 axis, we have determined that AMPK activates Arf6 upon glucose starvation and 5-aminoimidazole-4-carboxamide-1-β-D-ribofuranoside (AICAR) treatment. We have further identified the binding motif in the C-terminal regulatory domain of AMPK that is responsible for promoting Arf6 activation and, thus, inducing cell migration and invasion. These findings reveal a noncanonical role of AMPK in which its C-terminal regulatory domain serves as a GEF for Arf6 during glucose deprivation.

## Teaser

**Highlighted Article:** This study reveals a noncanonical role of AMP-activated kinase in which the α subunit C-terminal regulatory domain serves as a guanine-nucleotide-exchange factor for Arf6 during glucose deprivation.

## INTRODUCTION

Cells couple nutrient availability to signaling to support successful cell growth. During decreases in energy status upon starvation, AMP-activated protein kinase (AMPK), the major cellular energy sensor and metabolic switch, is activated to promote ATP production by upregulating catabolic pathways (Hardie et al., 2012). AMPK, a heterotrimeric complex, consists of a catalytic α subunit and two regulatory β and γ subunits. The α subunit AMPK catalytic core contains an N-terminal Ser/Thr kinase domain that is activated when a conserved residue, T172, in the activation loop is phosphorylated by its upstream kinase (Sanders et al., 2007). The C-terminal regulatory domain of AMPK α subunit binds to the β subunit. The γ subunit possess autoinhibitory properties that repress AMPK kinase activity (Chen et al., 2009). Emerging evidence has shown that AMPK plays a regulatory role in various tumors, acting as a tumor suppressor to reduce the aggressive behaviors of cancer cells (Zheng et al., 2013; Zhou et al., 2009). Impaired regulation of AMPK activity is thought to contribute to the induction of cancer cell invasion and migration (Chen et al., 2017; Yan et al., 2015). In contrast, AMPK activation also contributes to cancer progression through various cellular signaling pathways (Choudhury et al., 2014; Laderoute et al., 2014; Rios et al., 2013). However, the mechanism underlying AMPK-associated malignant phenotypes needs to be elucidated.

ADP-ribosylation factor 6 (Arf6), a small GTPase that is highly conserved across species, regulates vesicular trafficking and cytoskeletal reorganization (Boshans et al., 2000; Prigent et al., 2003). Arf6 is also involved in facilitating cancer cell migration and invasion (Hashimoto et al., 2004; Hongu et al., 2015; Palacios et al., 2001). Mechanistically, Arf6 acts through the recruitment of distinct effectors and the modulation of actin structure to induce downstream cellular events (Aikawa and Martin, 2003). Arf6 activation is regulated by guanine-nucleotide-exchange factors (GEFs), which facilitate the dissociation of guanosine diphosphate (GDP) and its replacement with guanosine triphosphate (GTP). Several GEFs, such as ARF nucleotide-binding site opener (ARNO, also known as CYTH2), that contain a Sec7 domain with nucleotide-exchange activity have been identified to activate Arf6 (Sztul et al., 2019). Although the Sec7 domain seems critical for promoting Arf6 activation, we have previously found that yeast Snf1 (the catalytic subunit of AMPK) acts as a noncanonical GEF to activate yeast Arf3, a homolog of mammalian Arf6, in response to glucose depletion (Hsu et al., 2015). However, whether mammalian AMPK can also act as a noncanonical GEF for Arf6 is unknown.

In this study, we demonstrate that AMPK, distinct from its canonical role as a kinase, acts as a GEF to activate Arf6. Activation of AMPK, either by treating with aminoimidazole-4-carboxamide-1-β-D-ribofuranoside (AICAR, an analog of adenosine monophosphate capable of stimulating AMPK kinase activity) or by glucose depletion, increases Arf6 activity and enhances cell migration and invasion. Our data reveal an unexpected role of the AMPK–Arf6 axis in mediating cancer cell motility under glucose deprivation, highlighting the role of AMPK in cancer cell progression to higher-grade malignancy.

## RESULTS AND DISCUSSION

### The C terminus of AMPK acts as a GEF for Arf6 *in vitro*

**Fig. 1.**
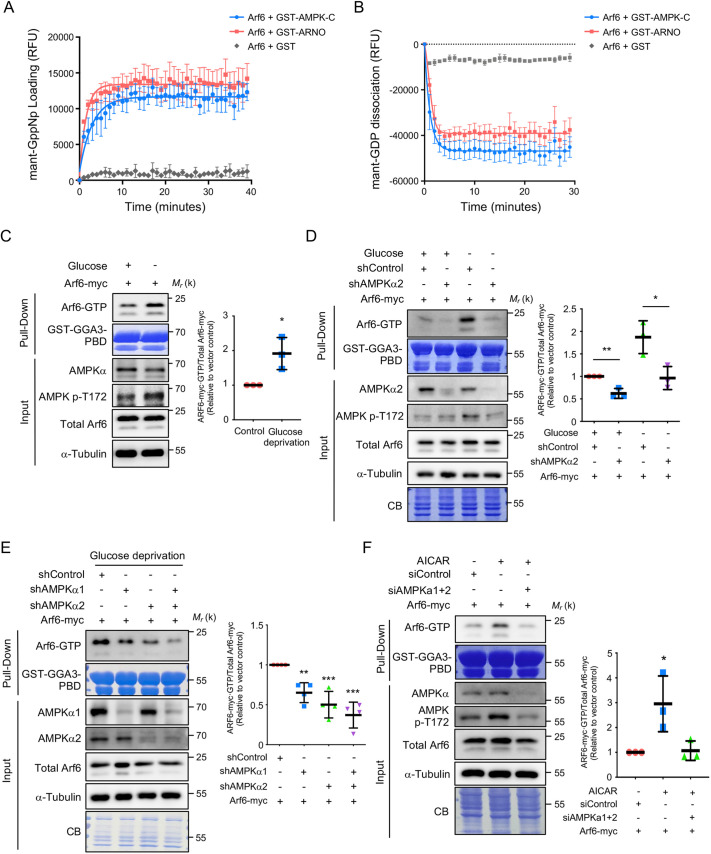
**AMPK activation increases the levels of Arf6-GTP.** (A,B) The C terminus of AMPK acts as a GEF for Arf6 *in vitro*. Assays of mant-GppNp binding to (A) and mant-GDP dissociation from (B) myristoylated Arf6 in the presence of GST-tagged recombinant AMPK-C or ARNO, or GST alone. The data are reported as the mean±s.d. of the percentages of bound mant-GppNp and dissociated mant-GDP, respectively; *n*=3 (RFU, relative fluorescence units). (C) Glucose depletion increases the level of Arf6-GTP. SKOV3 cells were subjected to glucose deprivation for 16 h. Cell lysis followed by a GST–GGa3-PBD pulldown assay was performed to examine the levels of Arf6-GTP. The scatter plot shows the mean±s.d.; *n*=3. *P≤0.05 (two-tailed unpaired Student's *t*-test). (D) AMPKα2 knockdown (shAMPKα2) reduces Arf6 activation. Pulldown assays with SKOV3 cells coexpressing the indicated plasmids and shRNAs were used to detect Arf6 activation (Arf6-GTP) under glucose-deprivation stress. The quantification of activity assessed by pulldown assays shown on the right is based on three biological replicates. The scatter plots show the mean±s.d.; *n*=3. **P≤0.01, ***P≤0.001 (one-way ANOVA with Dunnett's test). (E) Knockdown of both AMPKα1 (shAMPKα1) and AMPKα2 (shAMPKα2) reduces the activation of Arf6 under glucose deprivation. Pulldown assays with SKOV3 cells coexpressing the indicated plasmids and shRNAs were used to detect Arf6 activation (Arf6-GTP) under glucose deprivation. The scatter plot shows the mean±s.d.; *n*=4. **P≤0.01, ***P≤0.001 (one-way ANOVA with Dunnett's test). (F) AICAR activates AMPK and increases the level of Arf6-GTP in cancer cells. SKOV3 cells expressing the indicated myc-tagged Arf6 were treated with siRNAs against AMPKα1 and AMPKα2 (siAMPKa1+2) or control non-targeting siRNA (siControl) for 48 h followed by treatment with 0.5 mM AICAR for 6 h. Cell lysates were pulled down with GST–GGA3-PBD and immunoblotted to assess levels of Arf6-GTP. The scatter plot show the mean±s.d.; *n*=3. *P≤0.05 (one-way ANOVA with Dunnett’s test). In C–F, input blots represent 5% (C,F) or 2.5% (D,E) of the total lysate, and α-tubulin is shown as a loading control. CB, Coomassie Blue staining. Molecular mass (M_r_) is indicated in kDa.

We previously identified yeast Snf1 as a noncanonical GEF for the activation of yeast Arf3 through its C-terminal domain (Hsu et al., 2015). We sought to examine whether AMPK exhibits similar activity for Arf6 activation in mammalian cells. We first characterized the interaction between Arf6 and AMPK and found that the recombinant Arf6-T27N, a GTP binding-deficient mutant, directly interacted with a C-terminal fragment of AMPKα2 (AMPK-C) (Fig. S1A). AMPK-C also directly interacted with recombinant Arf6 preloaded with GDP but not GTPγS (Fig. S1B). Furthermore, the Arf6-T27N, but not the constitutively GTP-bound Arf6-Q67L, associated with AMPK. This interaction was reproduced by reciprocal immunoprecipitation (IP) using an anti-myc antibody (Fig. S1C). These results demonstrate that AMPK interacts directly with Arf6 in a GDP-dependent manner through its C-terminal domain. To test whether AMPK also possesses GEF activity for Arf6, we purified recombinant AMPKα2 C-terminal fragments to examine their GEF activity toward recombinant Arf6 *in vitro* and found that AMPK-C promoted GDP release from (Fig. 1A) and GTP loading of (Fig. 1B) Arf6, comparable to the effects of ARNO, a well-characterized Arf6 GEF. These data indicate that AMPK-C can stimulate the release of GDP to allow the binding of GTP to Arf6.

### AMPK activates Arf6 *in vivo*

Our previous data demonstrated that yeast Snf1 activates Arf3 in response to glucose depletion (Hsu et al., 2015). Thus, a potential role of glucose starvation would further support the function of AMPK as a GEF for Arf6. SKOV3 ovarian carcinoma cells are known to be highly sensitive to glucose deprivation because of the induction of AMPK activity (Priebe et al., 2011). Indeed, we observed an increase in the Arf6-GTP level under glucose depletion (Fig. 1C). We investigated the involvement of AMPK in glucose starvation-regulated Arf6 activation and demonstrated a reduction in the amount of active Arf6 in AMPKα2 (PRKAA2)-knockdown cells (Fig. 1D). We also assessed the relative contributions of two AMPK catalytic isoforms, AMPKα1 (PRKAA1) and AMPKα2, to the regulation of Arf6 activation. Knocking down either AMPKα1 or AMPKα2 in SKOV3 and OVCAR3 cells decreased the amount of active Arf6, and knockdown of both AMPKα1 and AMPKα2 resulted in a synergistic reduction in the Arf6-GTP level (Fig. 1E; Fig. S2A). Under normal glucose conditions, the active Arf6 also decreased when AMPKα1 and AMPKα2 were knocked down in SKOV3 or HEK 293T cells (Fig. S2B,C). These data suggest that glucose starvation induces AMPK activation to regulate Arf6 activity and both AMPK isoforms contribute to Arf6 activation under glucose deprivation.

AMPK can be efficiently activated by pharmacological perturbation using AICAR (Sun et al., 2007). AMPKα T172 phosphorylation (p-T172) was increased in cells treated with AICAR (Fig. 1F) Importantly, AICAR treatment was sufficient to activate Arf6, and siRNA targeting AMPKα1 and AMPKα2 prevented AICAR-induced Arf6 activation (Fig. 1F). Similar results were observed in AICAR-treated HEK 293T cells with AMPKα1 and AMPKα2 knockdown (Fig. S2D). These data indicate that AMPKα phosphorylation is correlated with Arf6 activation. Importantly, the AMPK activator AICAR not only enhanced the kinase activity of AMPK but also stimulated its GEF activity toward Arf6. Future studies will focus on whether AICAR-induced conformational changes in AMPK assist in the exposure of the GEF domain for nucleotide exchange on Arf6.

### AMPK activation of Arf6 does not require the AMPK kinase domain

**Fig. 2.**
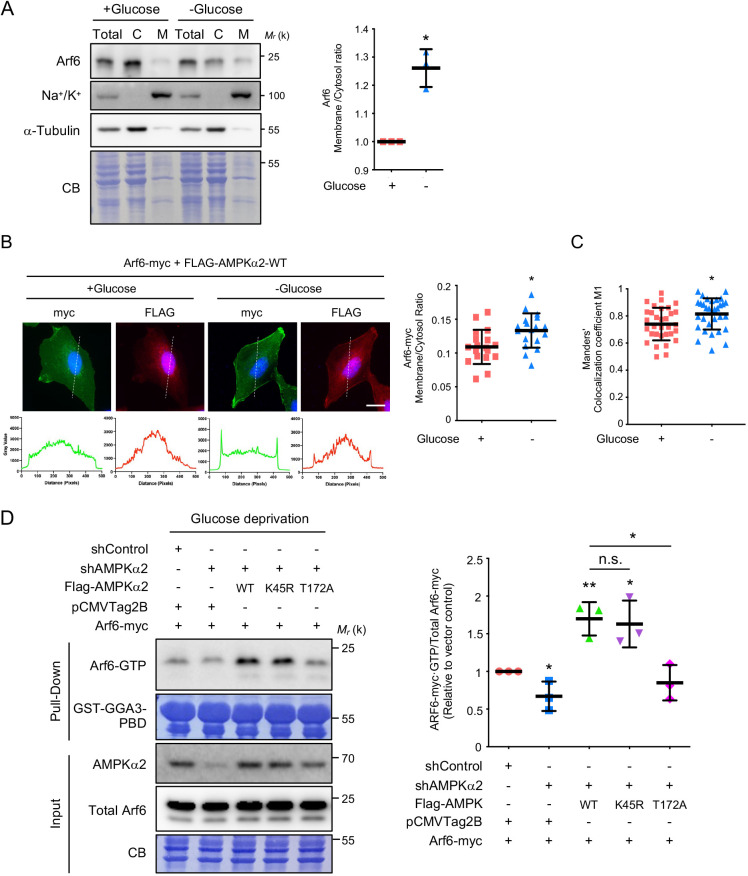
**Arf6 activation by AMPK is independent of AMPK kinase activity under glucose deprivation.** (A) Glucose deprivation redistributes cytosolic Arf6 to the PM. SKOV3 cells were treated as indicated and fractionated into cytosolic (C) and membrane (M) fractions. The relative distribution of Arf6 was assessed. α-tubulin marks the cytosol, and Na^+^/K^+^-ATPase marks the membrane. Quantification of membrane:cytosol Arf6 ratio is presented on the right as the mean±s.d. of *n*=3 experiments. *P≤0.05 (two-tailed unpaired Student's *t*-test). (B) Glucose deprivation induces the colocalization of Arf6 and AMPK on the PM. SKOV3 cells were cotransfected with Arf6–myc and FLAG–AMPKα2-WT, subjected to glucose deprivation for 16 h, and then stained with anti-myc and anti-FLAG antibodies, as indicated. Nuclei were stained using DAPI (blue). Linear profiles, as indicated by the dashed lines created using ImageJ software, are plotted beneath the images and show the PM and cytosolic localization of the fluorescent proteins across the cell. Quantification of the Arf6 membrane:cytosol localization ratio is presented as the mean±s.d. of *n*=18 cells. *P≤0.05 (two-tailed unpaired Student's *t*-test). Scale bar: 20 µm. (C) Quantification of Arf6 and AMPK colocalization coefficients on the PM. Arf6–myc and FLAG–AMPKα2-WT were expressed in SKOV3 cells with or without glucose, as indicated. The colocalization coefficients of AMPKα2 and Arf6 were calculated using imageJ. The data are presented as the mean±s.d. of *n*=32 cells. *P≤0.05 (two-tailed unpaired Student's *t*-test). (D) T172 phosphorylation but not AMPK kinase activity is required for AMPK-mediated Arf6 activation under glucose deprivation. Pulldown assays were performed on SKOV3 cells transfected with the indicated plasmids and shRNAs. The active GTP-bound form of Arf6 was pulled down using purified GST–GGA3-PBD. Interacting proteins bound to the GST beads were analyzed by immunoblotting. Input blots represent 2.5% of the total lysate. Right panel, quantitative analysis of active Arf6 for the indicated conditions. The data are reported relative to the empty vector control. The scatter plots shows the mean±s.d.; *n*=3. *P≤0.05; **P≤0.01; n.s., not significant (one-way ANOVA with Dunnett's test). In A and D, molecular mass (M_r_) is indicated in kDa. CB, Coomassie Blue staining.

Arf6 is known to be distributed between the cytosolic and membrane fractions, and active Arf6 localizes predominantly to the plasma membrane (PM) (Radhakrishna and Donaldson, 1997). Subcellular fractionation showed that glucose deprivation redistributed cytosolic Arf6 to the PM (Fig. 2A). Consistent with this result, Arf6 showed increased PM targeting and colocalized with AMPKα2 at the PM under glucose deprivation (Fig. 2B,C). These data indicate that AMPK acts upstream of Arf6 as a GEF to elevate the GTP-bound fraction of Arf6. Notably, the decreased level of Arf6-GTP in AMPKα2-knockdown cells was rescued by the expression of wild-type AMPKα2 (Fig. 2D). Consistent with results in yeast (Hsu et al., 2015), the expression of the kinase-dead mutant AMPKα2-K45R, but not the phosphorylation mutant AMPKα2-T172A, activated Arf6 (Fig. 2D). These data indicate that AMPK activation of Arf6 does not require its kinase domain, yet the phosphorylation on T172 of AMPKα is required for this activation process.

### AMPK activates Arf6 through its C-terminal regulatory domain

**Fig. 3.**
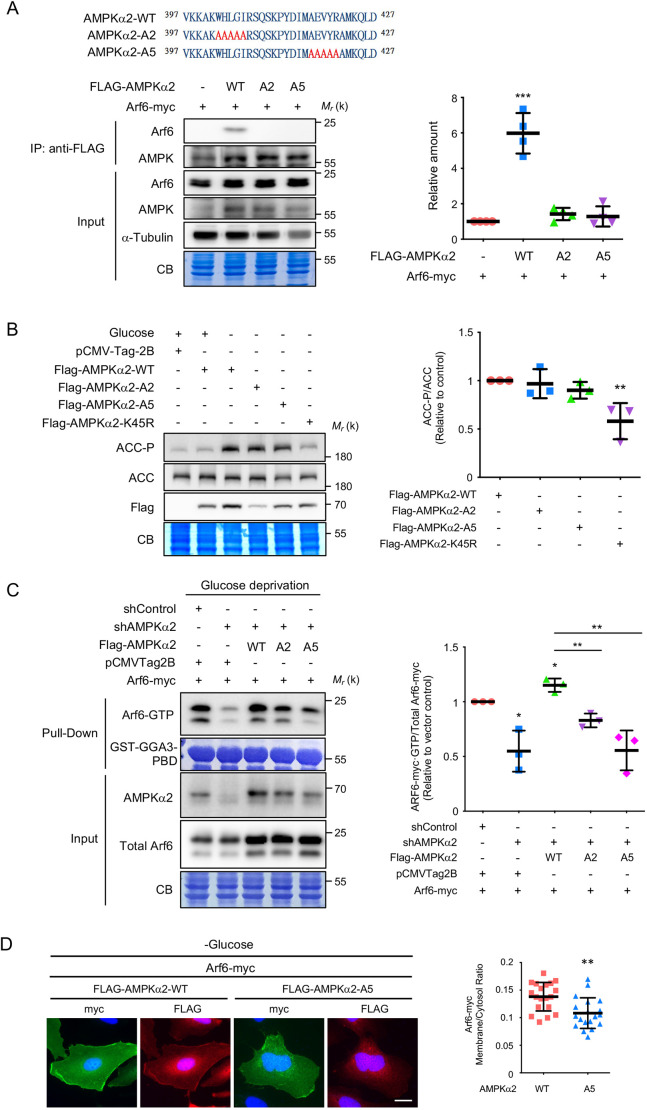
**The C terminus of AMPKα2 interacts with and activates Arf6.** (A) Top left: sequences of the C-terminal portions of AMPKα2, AMPKα2-A2 and AMPKα2-A5 (with amino acid residues indicated). Bottom left: interactions were verified by transfecting HEK 293T cells with the indicated plasmids, performing IP with an anti-FLAG antibody, and immunoblotting to detect Arf6. Input blots represent 2% of the total lysate, and α-tubulin is shown as a loading control. Right: scatter plot showing the mean±s.d. relative amount of Arf6 immunoprecipitated; *n*=4. **P≤0.01 (one-way ANOVA with Dunnett's test). (B) The AMPKα2-A2 and AMPKα2-A5 mutants do not affect the kinase activity of AMPK. HEK 293T cells were transfected with the indicated plasmids (pCMV-Tag-2b, empty vector control) for 24 h and then treated with glucose deprivation for 16 h. Cells were harvested, and the levels of ACC and phosphorylated ACC (ACC-P) were checked by western blotting. The scatter plot shows the mean±s.d. ACC phosphorylation; *n*=3. **P≤0.01 (one-way ANOVA with Dunnett's test) (C) AMPKα2-A2 and AMPKα2-A5 fail to rescue the reduced Arf6-GTP level in AMPKα2-knockdown cells. GST–GGA3-PBD pulldown assays were performed on AMPKα2-knockdown (shAMPKα2) SKOV3 cells with expression of indicated plasmids. Input blots represent 2.5% of the total lysate. Right, quantitative analysis of active Arf6 (Arf6-GTP). The scatter plot shows the mean±s.d.; *n*=3. *P≤0.05; **P≤0.01 (one-way ANOVA with Dunnett's test). In A–C, molecular mass (M_r_) is indicated in kDa. CB, Coomassie Blue staining. (D) Wild-type AMPKα2, but not the Arf6-binding defective mutant AMPKα2-A5, induces Arf6 PM localization under glucose deprivation. SKOV3 cells underwent AMPKα2 knockdown and were then cotransfected with Arf6–myc and FLAG–AMPKα2-WT or FLAG–AMPKα2-A5. Cells were subjected to glucose deprivation for 16 h and then stained with the indicated antibodies. Nuclei were stained using DAPI (blue). The membrane:cytosol ratio of Arf6 localization was quantified, and the data are presented as the mean±s.d. of *n*=19 cells. **P≤0.01 (two-tailed unpaired Student's *t*-test). Scale bar: 20 µm.

Structural biology analysis has revealed that the C-terminal regulatory domain of AMPKα2 is evolutionarily conserved, with several similar hydrophobic residues present from yeast to mammals (Crozet et al., 2014). We generated two mutants of AMPKα2 with substitution of these conserved hydrophobic residues with alanine: AMPKα2-A2 (amino acids 402–406) and AMPKα2-A5 (amino acids 417–421). We found that both AMPKα2-A2 and AMPKα2-A5 mutants were defective in binding to Arf6 in SKOV3 and HEK 293T cells (Fig. 3A; Fig. S2E). We further demonstrated that the phosphorylation of acetyl-CoA carboxylase 1 (ACC1), a well-known substrate of AMPK, was comparable in cells expressing either AMPKα2-A2 or AMPKα2-A5, compared with cells expressing wild-type AMPKα2 (AMPKα2-WT; Fig. 3B), suggesting that these mutations do not alter AMPK kinase activity. Moreover, compared to reconstitution of AMPKα2-WT, reconstitution of either AMPKα2-A2 or AMPKα2-A5 in AMPKα2-knockdown cells decreased the Arf6-GTP level (Fig. 3C). Moreover, AMPKα2-A5 was found to be defective in inducing Arf6 PM localization (Fig. 3D). These findings suggest that C-terminal hydrophobic residues of AMPKα2 interact with Arf6 to promote nucleotide exchange on Arf6.

### A critical role for AMPK-mediated Arf6 activation in cancer cell migration

**Fig. 4.**
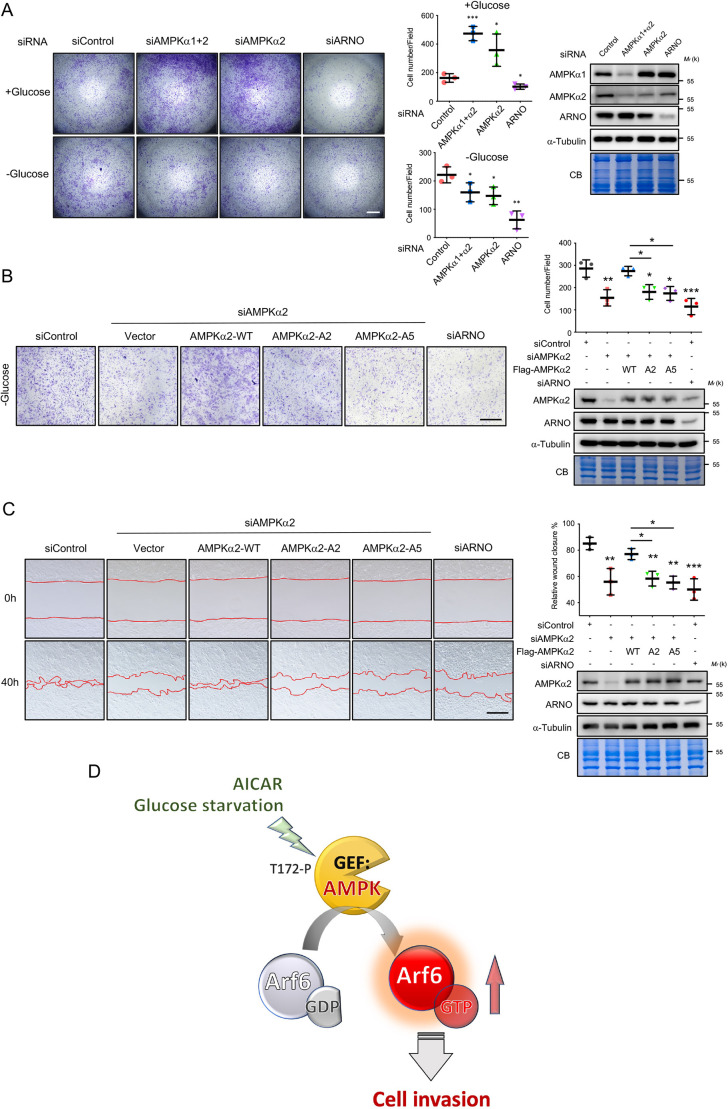
**The AMPK–Arf6 axis is necessary for glucose deprivation-induced cell invasion.** (A) AMPKα-knockdown enhances the cellular invasion ability under normal glucose conditions. Left: Transwell invasion assays, with or without glucose deprivation, were performed using SKOV3 cells after knockdown with the indicated siRNAs for 48 h. After 24 h of cell invasion, cells were stained with Crystal Violet for quantification. Middle: graphs showing the resulting quantification of cell invasion, presented as the mean number of cells per field of view. Right: levels of the indicated proteins were checked by immunoblotting and Coomassie Blue staining. (B) AMPKα2-A2 and AMPK α2-A5 fail to rescue the reduced invasion in AMPKα2-knockdown cells under glucose deprivation. Left: representative images of invasion assays, as described in A, of SKOV3 cells transfected with indicated plasmids and siRNAs. Top right: quantification of cell invasion for the indicated conditions, as described in A. Bottom right: levels of the indicated proteins were checked by immunoblotting. (C) AMPKα2-A2 and AMPKα2-A5 fail to rescue wound healing under glucose deprivation. Left: representative images of wound healing assays of SKOV3 cells transfected with indicated siRNAs and plasmids under glucose-deprivation conditions. Cells were imaged directly after wounding (0 h), and confluent monolayers were photographed at 40 h after wounding. Monolayer boundaries are outlined. Top right: quantification of wound closure for the indicated conditions. Bottom right: levels of the indicated proteins were checked by immunoblotting. The scatter plots in A–C show the mean±s.d; *n*=3. *P≤0.05; **P≤0.01; ***P≤0.001 (one-way ANOVA with Dunnett's test). For the western blotting in A–C, molecular mass (M_r_) is indicated in kDa. CB, Coomassie Blue staining. Scale bars: 250 μm in A; 200 μm in B,C. (D) A model of Arf6 activation by AMPK in response to glucose deprivation (T172-P, phosphorylation of AMPKα at T172). See the main text for details.

Activation and high expression of Arf6 correlate strongly with invasion and metastasis in several tumor types (Grossmann et al., 2013; Morishige et al., 2008). In addition, both tumor suppressive and oncogenic roles of AMPK have been reported (Kim et al., 2011; Laderoute et al., 2014). We tested whether interfering with the ability of AMPK to act on Arf6 by modulating its GTP-loading affects cancer cell invasion. Under normal and glucose-deprivation conditions, an invasion assay revealed that knockdown of Arf6 or of the Arf6-GEF ARNO reduced the invasion ability of SKOV3 cells (Fig. 4A; Fig. S3A). AMPKα-knockdown enhanced cell invasion under normal glucose conditions. Notably, AMPKα-knockdown significantly reduced the invasion ability of SKOV3 cells under glucose-deprivation conditions (Fig. 4A), suggesting that energy stress is involved in switching the role of AMPK in the modulation of cell invasion. This result was confirmed by using two different siRNA sequences targeting AMPKα2 (Fig. S3B). We then ectopically expressed AMPKα2-WT to rescue cell invasion in AMPKα2-knockdown SKOV3 and OVCAR3 cells under glucose-deprivation. Importantly, expression of Arf6-binding detective mutants of AMPKα2, AMPKα2-A2 and AMPKα2-A5, failed to restore this reduction in invasion (Fig. 4B; Fig. S3C). In addition, a decrease in wound closure was observed when AMPKα2-A2 and AMPKα2-A5 were used to rescue AMPKα2-knockdown cells under glucose deprivation (Fig. 4C; Fig. S3D). These data indicate that Arf6 activation by AMPK is critically important to promote cancer cell migration specifically under glucose-deprivation conditions. A recent study has reported that glucose starvation induces the expression of MMP9 and phosphorylation of AMPK, which in turn promotes cell migration (Endo et al., 2018). These results support the theory that the impact of AMPK in the modulation of cell invasion under glucose deprivation is a cell context-dependent process.

Most previous data suggest that AMPK could be a tumor suppressor due to functional loss of an AMPK upstream kinase, liver kinase B1 (LKB1, also known as STK11) (Shackelford and Shaw, 2009). However, recent accumulated data also indicate that AMPK might be a protumorigenic regulator. For instance, AMPK activity has been found to be necessary for the induction of epithelial–mesenchymal transition in cancer cells by hypoxia and TGFβ treatment (Saxena et al., 2018)*.* AMPKα1*-*knockout cancer cells also exhibit the attenuation of cancer cell migration and invasion induced by TLR4 simulation (Kim et al., 2020). In addition, AMPK activation by lysophosphatidic acid (LPA) promotes the metastasis of SKOV3 cells (Kim et al., 2011). Energy stress in cells due to glucose depletion appears to be a driving force of cancer cell invasiveness and migration (Endo et al., 2018). Specifically, AMPK is usually activated when T172 of the α subunit is phosphorylated by an upstream kinase in response to glucose starvation. We also demonstrate that the AMPK activator AICAR activates Arf6, suggesting a novel mechanism for nucleotide exchange of Arf small GTPase. We found that the oncogenic function of AMPK may be correlated with its downstream substrate Arf6, as under these conditions, AMPK acts as a GEF rather than a kinase of Arf6 (Fig. 4D). The paradox between the protective role of AMPK in metabolic adaptation and its tumorigenic role in promoting cancer cell motility during energy imbalance needs further investigation.

In this study, we reveal that AMPK functions as a GEF to activate Arf6, leading to the redistribution of cytosolic Arf6 to the PM, which then promotes cancer cell migration and invasion. This study shows a fundamentally different role of AMPK as a GEF rather than a kinase of its downstream substrates. Thus, AMPK, the major cellular energy sensor, is a potential anticancer target, indicating that inhibiting the GEF activity of AMPK may decrease cancer cell motility in response to glucose starvation.

## MATERIALS AND METHODS

### Plasmids and cell culture

Human cervical carcinoma HeLa cells were purchased from the American Type Culture Collection (ATCC; Manassas, VA, USA). The human serous cystadenocarcinoma cell line SKOV3 was kindly provided by Dr Lin-Hung Wei, National Taiwan University Hospital, Taipei City, Taiwan. HEK 293T cells were provided by Dr Tzuu-Shuh Jou, National Taiwan University, Taipei City, Taiwan. HeLa, SKOV3 and HEK 293T cells were cultured in high-glucose Dulbecco's modified Eagle's medium (HG-DMEM; SH30007.02, HyClone) supplemented with 10% fetal bovine serum (FBS; HyClone) at 37°C in an atmosphere containing 5% CO_2_. OVCAR3 cells were purchased from Bioresource Collection and Research Center (BCRC; Hsinchu, Taiwan) and cultured in RPMI 1640 medium (SH30011.02, HyClone) with 10% FBS. Cells were transiently transfected with the indicated plasmids or siRNAs using Lipofectamine 2000 transfection reagent (Invitrogen) according to the manufacturer's instructions. The shControl (ASN0000000002) and shAMPK (TRCN002168) lentiviral pLKO.1 plasmids were provided by the National RNAi Core Facility of Academia Sinica, Taipei, Taiwan. The AMPKα2-K45R and AMPKα2-T172A plasmids were gifts from Dr Sheng-Chung Lee, National Taiwan University, Taipei, Taiwan. For expression as a GST fusion protein in *Escherichia coli* and an N-terminal FLAG fusion protein in mammalian cells, ARNO was cloned into the pGEX-4T and pCMV-Tag2 vectors as described previously (Li et al., 2007). The GGA3-PBD (amino acids 1–316) cDNA was amplified from a HeLa cell cDNA pool (Li et al., 2007). For AICAR treatment, cells were treated with 0.5 mM AICAR (Millipore, #123040) for 6 h.

### *In vitro* binding assay

*E. coli* strain BL21 was transformed with plasmids. To express N-terminally His-tagged Arf6, the Arf6 (WT or d13N-T27N) fragments were cloned into pET32a (Biomedical Resource Core at the First Core Labs, National Taiwan University College of Medicine). After cells were induced with 0.5 mM isopropyl β-D-1-thiogalactopyranoside (IPTG) at 37°C for 3 h, cells were pelleted by centrifugation, suspended in lysis buffer [20 mM Tris-HCl (pH 7.9), 5 mM imidazole, 500 mM NaCl, 10% glycerol, 0.5 mg/ml lysozyme and 1× protease inhibitor (1 μg/ml aprotinin, 1 mg/ml benzamidine, 1 μg/ml pepstatin A, 1 μg/ml antipain, 50 μg/ml Nα-Tosyl-L-lysine chloromethyl ketone hydrochloride and 1 μg/ml leupeptin)] and incubated on ice for 30 min, lysed with a nitrogen bomb at a pressure of 1500 pounds per square inch, then the debris was removed by centrifugation (14,000 ***g***, 30 min). The resulting supernatant was bound to glutathione S-transferase (GST) fusion proteins, and His-tagged proteins were purified using glutathione-Sepharose 4B (GE Healthcare Amersham, Piscataway, NJ) and nickel affinity resin (Qiagen, Valencia, CA), respectively. In the pulldown assays, GST and GST–AMPK-C (amino acids 291–552 of AMPKα2) were immobilized on glutathione beads and incubated with His–Arf6 (WT or T27N) in binding buffer containing 25 mM Tris-HCl (pH 7.5), 150 mM NaCl, 0.5% Nonidet P-40 (NP-40), 5 mM MgCl_2_ and 1× protease inhibitor for 1 h at 4°C. Then, the beads were washed three times with 1 ml of binding buffer. Next, bound proteins were analyzed by western blotting using anti-His monoclonal antibodies (BD Biosciences, La Jolla, CA; no. 552565; 1:5000). For the GTP and GDP loading assay, wild-type Arf6 was loaded with either GDP or GTP by incubation with 1 mM GDP or nonhydrolyzable GTPγS, respectively, at 30°C for 15 min. The binding assay was then performed as described above.

### *In vitro* GEF activity assays

The *in vitro* GEF activity assays were performed as previously described (Stalder et al., 2011). Nucleotide exchange was monitored as the fluorescence change of mant-GDP (N-methylanthraniloyl guanosine 5-diphosphate) dissociation from myr-Arf6 and mant-GppNp [N-methylanthraniloyl guanosine 5-(β,γ-imido)-triphosphate] loading to myr-Arf6. Recombinant C-terminal His-tagged Arf6 was myristoylated by co-expressing with yeast N-myristoyltransferase in *E. coli* then purified using Ni^2+^-NTA agarose (Qiagen, 1018244), as previously described (Chavrier and Franco, 2001; Stalder et al., 2011). For the mant-GDP dissociation assay, reactions were initiated by addition of 1 μM myr-Arf6–mant-GDP, and nucleotide exchange was catalyzed by the addition of 100 µM GppCp and 100 nM GST, GST–AMPK-C, or GST–ARNO. For the mant-GppNp loading assay, 1 μM myr-Arf6 and 10 µM mant-GppNp with the addition of 100 nM GST, GST–AMPK-C or GST–ARNO. All reactions were carried out at 30°C in the presence of 100 µM liposomes composed of 45% phosphatidylcholine (PC), 20% phosphatidylethanolamine (PE), 30% phosphatidylserine (PS), and 5% phosphatidylinositol (4,5)-bisphosphate (PIP_2_) in exchange buffer containing 20 mM HEPES (pH 7.5), 100 mM KCl, 1 mM DTT, 2 mM EDTA and 1 mM MgCl_2_, using an excitation wavelength of 360 nm and emission measured at 440 nm.

### Small G protein activity pulldown assay

The Arf6 GTPase was trapped in the GTP-bound state with a Golgi-associated gamma-adaptin ear-containing ARF binding protein 3 (GST–GGA3-PBD) fusion protein containing the VHS- and ARF-binding domains of GGA3 (amino acids 1–316) coupled to Sepharose beads. For the Arf6 activity assay, SKOV3 cells were transfected with the indicated plasmid or knockdown lentiviral plasmid. For glucose deprivation treatment, SKOV3 cells were prewashed with phosphate-buffered saline (PBS) three times. Then, glucose-free DMEM (Gibco, 11966025) or glucose-free RPMI (Gibco, 11879020) containing 10% FBS was added for 16 h. Cells were lysed in pulldown buffer containing 25 mM Tris-HCl (pH 7.5), 150 mM NaCl, 0.5% NP-40, 5 mM MgCl_2_, and 1× protease inhibitor. A 40 μg sample of GST–GGA3-PBD was incubated with 1 mg of cell lysate for 1 h at 4°C. The GST beads were then washed three times with pulldown buffer. Proteins were eluted with protein loading buffer and analyzed by immunoblotting. Equal amounts of GST beads were determined by Coomassie Blue staining.

### Co-immunoprecipitation

HEK 293 T cells were seeded in 10 cm dishes and transiently transfected with the indicated plasmid using Lipofectamine 2000 transfection reagent. After 16 h of transfection, the cells were lysed with IP buffer [50 mM Tris-HCl (pH 7.4), 150 mM NaCl, 1% NP-40, 1× protease inhibitor], and the lysate was centrifuged at 14,000 ***g*** for 10 min at 4°C. The supernatants were incubated with a monoclonal anti-myc antibody (Covance, MMS-150R) or anti-Flag antibody (Sigma, F3165) for 2 h at 4°C. Then, the cells were incubated with Protein A Sepharose beads (Merck, GE17-5280-01) for 1 h at 4°C. The beads were washed three times with wash buffer. The co-immunoprecipitated proteins were eluted with sample buffer, boiled and analyzed via western blotting.

### Transwell invasion assay

The Transwell invasion assay was performed using Boyden transwell chambers with 8 µm pore size polycarbonate membranes (Corning, Costar, #3464). Transwell membranes were precoated with 70 µl of Matrigel (diluted 1:5 with culture medium; Corning, #356234) in a 24-well Transwell plate, and the Matrigel was allowed to solidify at 37°C for 1 h. After transfection of the indicated plasmids, 6×10^4^ cells in serum-free DMEM containing specific glucose concentrations were seeded into the prepared upper Transwell chambers, and 500 μl of DMEM supplemented with 10% FBS was added to the lower chambers. The 24-well plate was then incubated at 37°C in 5% CO_2_ for 24 h. Each assay was performed in duplicate. Cells were fixed with 4% paraformaldehyde in PBS for 15 min at room temperature. Noninvaded cells on the upper surface of the membranes were removed with a cotton swab, and invaded cells attached to the lower surface were stained with 1% Crystal Violet for 15 min. After washing, the membranes were air dried, and the invaded cells were imaged using a phase-contrast microscope (Nikon, Eclipse TS-100) connected to an imaging system (Nikon, DS-5 M), using a 100× objective. The invaded cells in at least three fields per well were counted using ImageJ software.

### Total protein extraction and immunoblot analysis

Western blotting was performed as described previously (Chen et al., 2020). After washing, the membranes were developed using an enhanced chemiluminescence system (Millipore). α-Tubulin was used as the internal control for protein loading. GST-tagged proteins were visualized by Coomassie Blue staining. Anti-α-tubulin (1:5000, T5168; Sigma-Aldrich), anti-AMPKα1 (1:1000, 2795S; Cell Signaling Technology), anti-AMPKα2 (1:1000, 2757S; Cell Signaling Technology), anti-CYTH2/ARNO (1:1000, H00009266; Abnova), anti-Arf6 (1:1000, #sc-7971, Santa Cruz Biotechnology), anti-AMPKα-pT172 (1:1000, 2535S; Cell Signaling Technology), anti-myc (1:1000, MMS-150R; Covance), anti-FLAG M2 (1:1000, F-3165; Sigma-Aldrich) anti-AMPKα (1:1000, 2532; Cell Signaling Technology), anti-ACC (1:1000, 3662; Cell Signaling Technology), anti-ACC-P (1:1000, 3661,Cell Signaling Technology) and anti-Na^+^/K^+^-ATPase (1:1000, sc-21712; Santa Cruz Biotechnology) antibodies were used.

### Immunofluorescence staining

The cells were fixed with 4% paraformaldehyde in PBS for 15 min. After washing with PBS, the cells were permeabilized with 0.01% Triton X-100 in PBS at room temperature for 5 min and blocked with 0.5% BSA in PBS. The following primary antibodies were diluted in blocking buffer at a 1:200 ratio: anti-myc (MMS-150R; Covance) and anti-FLAG M2 (F-3165; Sigma-Aldrich). Alexa Fluor 594 (A-11012; Invitrogen; anti-rabbit)- and Alexa Fluor 488 (A-11001; Invitrogen; anti-mouse)-conjugated secondary antibodies were used at a dilution of 1:1000. Images were acquired using an Axioplan microscope (Carl Zeiss, Inc.). Quantification of the membrane-targeting PM:cytoplasm ratio followed a previously published method (Chen et al., 2020).

### Wound healing assay

For the wound healing cell migration assay, cells were seeded in culture medium in six-well plates at a density of 8×10^5^ cells per well under glucose deprivation conditions. The layer of confluent cells was scratched using a fine pipette tip, washed twice with medium, and observed using a microscope (Carl Zeiss) after 22 h or 40 h. The cell migration capacity was determined using ImageJ software.

### Cell fractionation assay

SKOV3 cells (1×10^7^) were collected and washed twice with PBS and were then resuspended in 400 ml of buffer that consisted of 25 mM HEPES (pH 7.4), 2.5 mM MgCl_2_, 250 mM sucrose and 1× protease inhibitor. Cells were homogenized by passing through a 30-gauge needle 20 times. In general, 90–95% of nuclei were released from the cells, as determined by microscopy. The resulting homogenate was centrifuged at 100 ***g*** for 10 min to obtain the postnuclear supernatant. This supernatant was further centrifuged at 22,000 ***g*** for 1 h to obtain the cytosolic (supernatant) and membrane (pellet) fractions. Equal amounts of both fractions were analyzed by western blotting.

### Lentiviral knockdown

AMPKα1 and AMPKα2 knockdown by lentiviral shRNA silencing was performed by using packaging plasmids to produce lentiviruses containing pLKO.1-GFP-shRNA vectors (RNAi Core, Academia Sinica, Taiwan). After 24 h of infection, cells were selected with puromycin for 3 days. Green fluorescent protein (GFP) was used as a reporter for viral infection, and GFP-positive cells were evaluated to determine the infection efficiency. The sequences of the siRNAs and shRNAs used in this study are listed in Table S1.

### Statistical analysis

All data are expressed as the mean±s.d. values, and *P* values were calculated by two-tailed unpaired Student's *t*-test or one-way analysis of variance (ANOVA) followed by Dunnett's post hoc multiple comparison test using Excel or Prism 8. Significant differences (*P≤0.05; **P≤0.01; ***P≤0.001) are indicated. Each independent *in vitro* experiment included three biological replicates. Error bars reflect independent experiments.

## Supplementary Material

10.1242/joces.259609_sup1 Supplementary information
