# Supplementary material for: AMPK promotes Arf6 activation in a kinase-independent manner upon glucose starvation
Source: J Cell Sci. 2022 Sep 14;135(18):jcs259609. doi: 10.1242/jcs.259609 (PMC9584350; doi:10.1242/jcs.259609)
Supplement: Supplementary information [file joces-135-259609-s1.pdf]

Sup. Fig. S1.

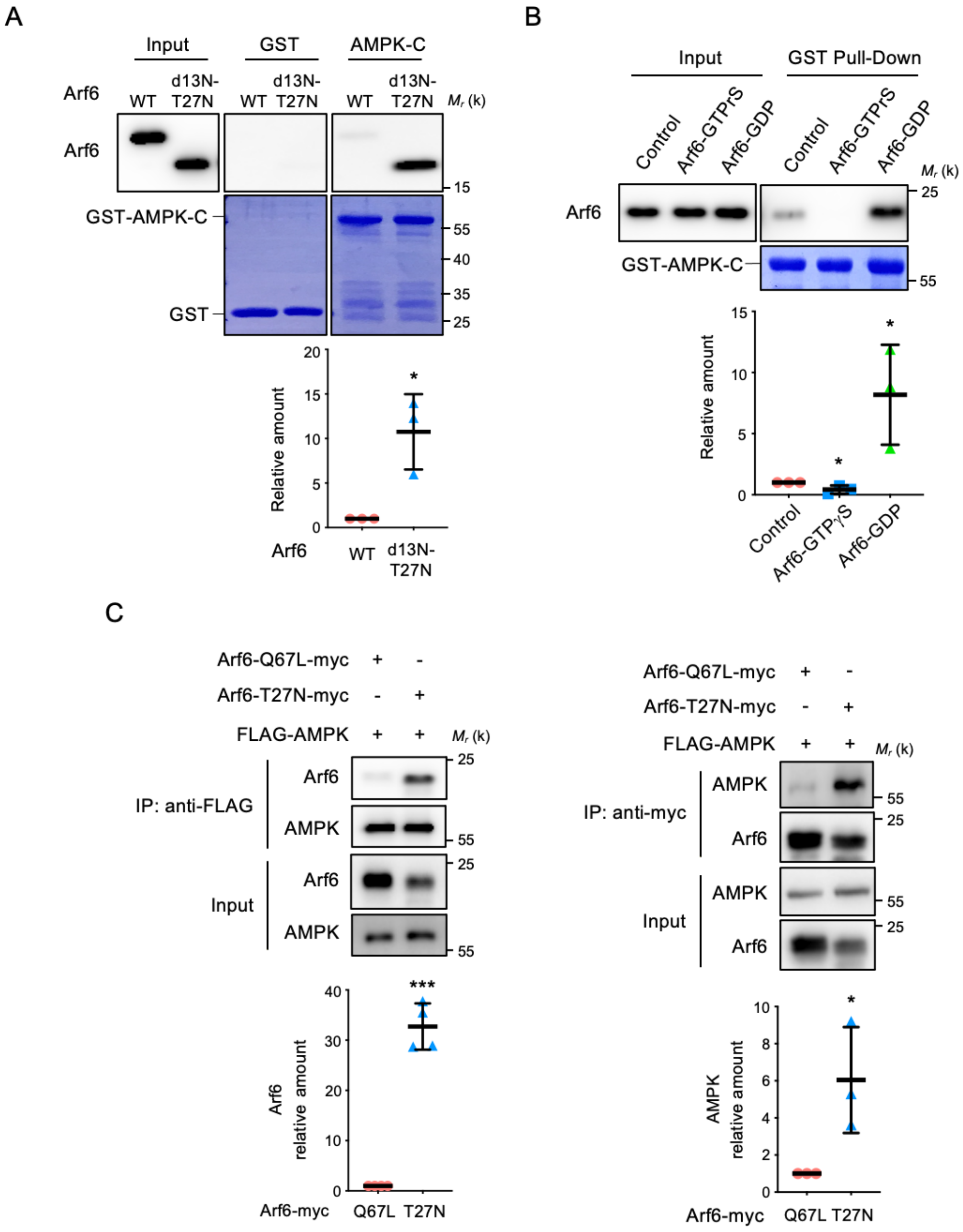

Fig. S1. AMPK binds to the inactive form of Arf6 (Arf6-GDP)

A) *In vitro* binding assay to evaluate the direct interaction between recombinant AMPK-C and Arf6. GST-AMPK-C purified from *E. coli* was incubated with recombinant His-Arf6^{WT} and His-Arf6^{T27N}-d13N for 1 h. Proteins were pulled down with glutathione-Sepharose beads and

Journal of Cell Science • Supplementary information

visualized by immunoblotting with an anti-Arf6 antibody. The scatter plots show the mean ± s.d. values; n=3. *P≤0.05 (Student's t-test).

B) Recombinant His-Arf6 was purified from *E. coli* and loaded with GTPγS or GDP. GST-AMPK-C pulldown of Arf6 in the nucleotide-free (Control), GTP-, or GDP-bound state. Proteins were separated by SDS-PAGE and visualized by Coomassie Blue staining. The scatter plots show the mean ± s.d. values; n=3. *P≤0.05. (one-way ANOVA with Dunnett's test).

C) Coimmunoprecipitation assays confirmed the interaction between AMPK and Arf-6. 293T cells cotransfected with FLAG-AMPK and Arf6-myc plasmids were lysed and subjected to IP with anti-FLAG antibodies and anti-myc antibodies, respectively. The IP complexes were then bound to Protein-A agarose beads, and the bound proteins were subjected to immunoblotting with antibodies as indicated. The scatter plots show the mean ± s.d.; n=4. *P≤0.05, ***P≤0.001 (Student's t-test).

Journal of Cell Science • Supplementary information

Sup. Fig. S2.

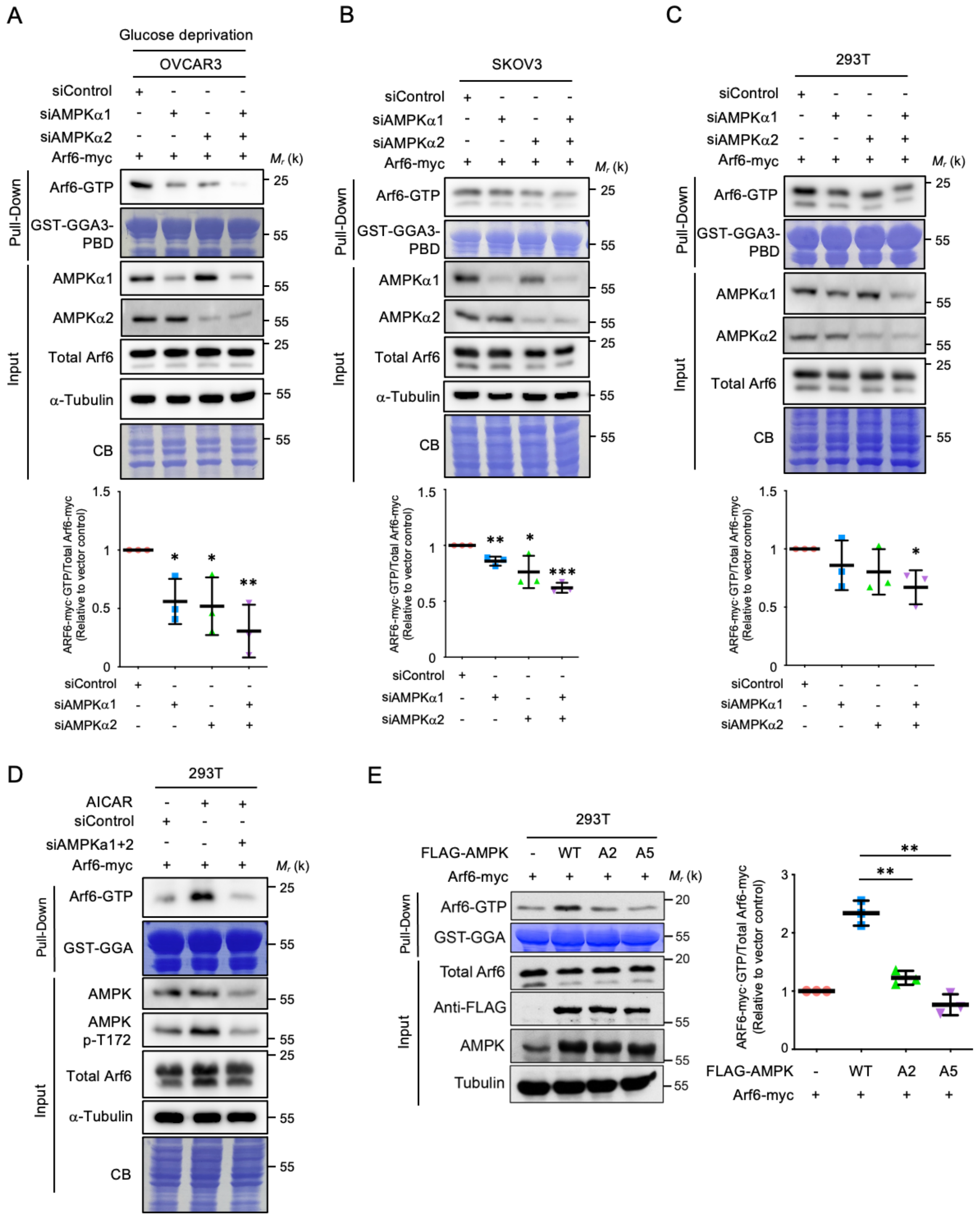

Fig. S2. AMPK-Arf6 axis in normal glucose conditions.

Journal of Cell Science • Supplementary information

A) Loss of AMPKα1 and α2 reduced Arf6 activation under glucose-deprivation in OVCAR3. Pulldown assays with OVCAR3 cells co-expressing the indicated plasmids or siRNAs were used to detect Arf6 activation under glucose-deprivation. The scatter plots show the mean ± SD values; n=3. *P≤0.05, **P≤0.01 (one-way ANOVA with Dunnett's test).

B-C) Reduction of Arf6 activity in both SKOV3 and 293T cells with co-knockdown of AMPKα1 and AMPKα2 under normal glucose conditions. Pulldown assays with cells co-expressing the indicated plasmids or siRNAs were used to detect Arf6 activation. The scatter plots show the mean ± s.d. values; n=4. *P≤0.05, **P≤0.01, ***P≤0.001 (one-way ANOVA with Dunnett's test).

D) AICAR activates AMPK and increases the level of Arf6-GTP in cancer cells. 293T cells were treated with siAMPKα1 and siAMPKα2 for 48 h followed by exposed to 0.5 mM AICAR for 6 h. Cell lysates were pulled down with GST-GGA3-PBD and immunoblotted for Arf6-GTP.

E) AMPK-A2 and AMPK-A5 failed to increase Arf6-GTP levels in 293T cells. Pulldown assays were performed on 293T cells expressing AMPKα2-WT, AMPK α2-A2 and AMPKα2-A5, as indicated. Right, quantitative analysis of active Arf6. The scatter plots show the mean ± s.d. values; n=3. **P≤0.01 (one-way ANOVA with Dunnett's test).

Journal of Cell Science • Supplementary information

Sup. Fig. S3.

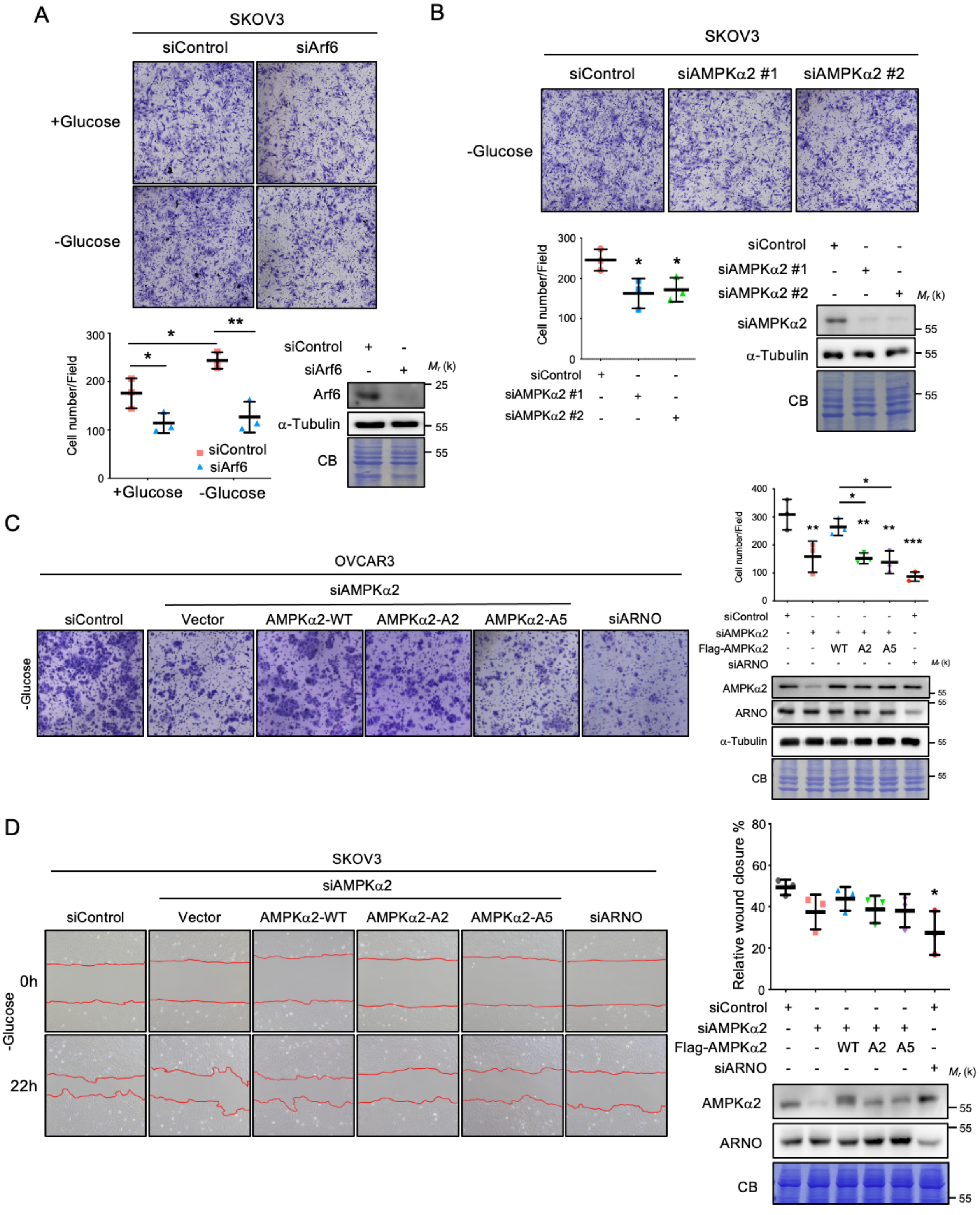

Fig. S3. Loss of the AMPK-Arf6 axis inhibits cancer cell invasion.

Journal of Cell Science • Supplementary information

A) Knockdown of Arf6 decreases the cell invasion ability. Transwell invasion assays were performed with SKOV3 cells after the indicated transfection and with or without glucose. The cells were stained and harvested after 24 h of invasion. Protein expression levels were checked by immunoblotting. *$P \leq 0.05$, **$P \leq 0.01$ (one-way ANOVA with Dunnett's test).

B) Knockdown of AMPK with a second siRNA also showed decreased invasion ability under glucose deprivation. Transwell invasion assays were performed with SKOV3 cells after knockdown with different siRNAs for 48 h. *$P \leq 0.05$ (one-way ANOVA with Dunnett's test).

C) AMPK1-WT, but not AMPK-A2 and A5, restored the cell migration defect in OVCAR3 cells treated with siRNA against AMPKα2. The scatter plots show the mean ± s.d. values; n=3. *$P \leq 0.05$, **$P \leq 0.01$, ***$P \leq 0.001$ (one-way ANOVA with Dunnett's test).

D) Wound healing in SKOV3 cells with the indicated genetic perturbation at 22 h. SKOV3 cells were transfected with the indicated siRNAs and plasmids under glucose deprivation. Confluent monolayers were photographed at 22 h after wounding. The scatter plots show the mean ± s.d. values; n=3. *$P \leq 0.05$ (one-way ANOVA with Dunnett's test).

Journal of Cell Science • Supplementary information

Table S1. Information on siRNA and shRNA oligonucleotides

**Target Gene**	**Cat. No.**	**siRNA Sequence**	**Company**
siAMPK (α1+α2)		AUGAUGUCAGAUGGUGAAUUUUU	Dharmacon
siAMPKα2 #1		GAGCAUGUACCUACGUUAUUUUU	Dharmacon
siAMPKα2 #2		GGAAGGUAGUGAAUGCAUAUU	Dharmacon
siARNO	SI00061299S1		QIAGEN
**Target Gene**		**shRNA Target Sequence**	
shControl	ASN0000000002	N/A (Scramble)	National RNAi Core Facility of Academia Sinica
shAMPKα1	NM_006251.5	GAAGGTTGTAAACCCATATTA	National RNAi Core Facility of Academia Sinica
shAMPKα2	NM_006252	GAGCATGTACCTACGTTATTT	National RNAi Core Facility of Academia Sinica

Journal of Cell Science • Supplementary information
