# Supplementary material for: AMPK promotes Arf6 activation in a kinase-independent manner upon glucose starvation
Source: J Cell Sci. 2022 Sep 14;135(18):jcs259609. doi: 10.1242/jcs.259609 (PMC9584350; doi:10.1242/jcs.259609)
Supplement: Supplementary file 1 [file joces-135-259609_review_history.pdf]

AMPK promotes Arf6 activation in a kinase-independent manner upon glucose starvation

Kuan-Jung Chen, Jia-Wei Hsu and Fang-Jen S. Lee
DOI: 10.1242/jcs.259609

Editor: Michael Way

Review timeline
Original submission: 22 November 2021
Editorial decision: 29 December 2021
First revision received: 6 May 2022
Editorial decision: 14 June 2022
Second revision received: 9 August 2022
Accepted: 10 August 2022

Original submission

First decision letter

MS ID#: JOCES/2021/259609

MS TITLE: AMPK promotes Arf6 activation in a kinase-independent manner upon energy deprivation

AUTHORS: Kuan Jung S Chen, Jia-Wei Hsu, and Fang-Jen S Lee
ARTICLE TYPE: Short Report

We have now reached a decision on the above manuscript.

To see the reviewers' reports and a copy of this decision letter, please go to: https://submit-jcs.biologists.org and click on the 'Manuscripts with Decisions' queue in the Author Area. (Corresponding author only has access to reviews.)

As you will see, the three reviewers are positive but raise a number of substantial criticisms that prevent me from accepting the paper at this stage. They suggest, however, that a revised version might prove acceptable, if you can address their concerns. If you think that you can deal satisfactorily with the criticisms on revision, I would be pleased to see a revised manuscript. We would then return it to the reviewers.

We are aware that you may be experiencing disruption to the normal running of your lab that makes experimental revisions challenging. If it would be helpful, we encourage you to contact us to discuss your revision in greater detail. Please send us a point-by-point response indicating where you are able to address concerns raised (either experimentally or by changes to the text) and where you will not be able to do so within the normal timeframe of a revision. We will then provide further guidance. Please also note that we are happy to extend revision timeframes as necessary.

Please ensure that you clearly highlight all changes made in the revised manuscript. Please avoid using 'Tracked changes' in Word files as these are lost in PDF conversion.

I should be grateful if you would also provide a point-by-point response detailing how you have dealt with the points raised by the reviewers in the 'Response to Reviewers' box. Please attend to

© 2022. Published by The Company of Biologists under the terms of the Creative Commons Attribution License (https://creativecommons.org/licenses/by/4.0/).

all of the reviewers' comments. If you do not agree with any of their criticisms or suggestions please explain clearly why this is so.

Reviewer 1

Advance summary and potential significance to field

This manuscript is an excellent, interesting and important study that reports a new guanine nucleotide exchange factor (activator) for the Arf family of small G proteins that is implicated in cancer cell migration and invasion. To date, only one major family of exchange factors for Arf family proteins has been identified, the Sec7 domain family. AMPK, which is a key regulator of cellular metabolism, is identified in this study as an exchange factor for mammalian Arf6, which is important for Arf6 capacity to promote cell migration and invasion. This group previously identified the yeast orthologue of AMPK as a GEF for Arf proteins in yeast, and here show that AMPK is an evolutionarily conserved activator of Arf proteins, which activates Arf6 in mammalian cells upon energy deprivation. Arf6 is an important regulator of membrane trafficking and cytoskeleton dynamics at the plasma membrane, and promotes cancer cell migration and invasiveness. In this study, the authors very clearly and convincingly demonstrate that the C-terminal region of the catalytic subunit of AMPK stimulates nucleotide exchange on Arf6 both in vitro and in vivo. Experiments in vivo were carried out using HEK 293T cells, as well as ovarian cancer cells (SKOV3) which are sensitive to glucose deprivation through induction of AMPK activity. The authors show that glucose starvation and AICAR activation of AMPK increases Arf6-GTP levels in SKOV3 cells, whereas knockdown of AMPKalpha1 and AMPKalpha2 leads to a decreased level of cellular Arf6-GTP.
Interestingly, the kinase activity of AMPK is not required for Arf6 activation but phosphorylation of Thr172 in the activation loop is required. The authors also show that in SKOV3 cancer cells, upon glucose starvation, invasiveness and cell migration are reduced upon AMPK siRNA treatment. These effects are rescued by wild type AMPK, but not by the C-terminal mutants that fail to activate Arf6.
In a supplementary figure, the authors show that AMPK binds to the GDP-bound inactive form of Arf6, both in pulldown experiments in cells and in vitro using purified proteins, as predicted for an exchange factor. The quality of the experimental data is excellent, and I only have minor comments.

Comments for the author

Detailed comments:
1. On page 8, first paragraph, the authors use the term “GGA3-PBD (1-316 a.a.)”, without defining “PBD”. Is it the same as the GGA3 portion of the fusion protein defined on page 9: “Golgi-associated gamma-adaptin ear-containing the ARF binding protein 3-GST (GGA3-GST) fusion protein”. In the figure legends, the GGA3 pull-down experiments are indicated as being performed by “GST-GGA3”, with “GST-GGA3-PBD” used in the figure panels. The same nomenclature should be used throughout, and defined clearly the first time the construct is described.
2. In Fig 1, why is there only one band in the Coomassie gel for GGA3-PBD in part C, but two bands in part E. I am assuming it is because the gels used were different, or the run times were different, but it would be good to explain this difference.
3. At the top of page 6, the authors state “These data indicate that AMPK activates Arf6 in a kinase-independent manner and that phosphorylation on Thr172 of AMPK is required for this activation process.”
Presumably the phosphorylation of AMPK Thr172 requires kinase activity, although it is clearly not an autophosphorylation. It would be better to say something like “AMPK activation of Arf6 does not require its kinase domain”.
4. Figure 2B. The authors state “Linear profiles, as indicated by the dashed lines created using ImageJ software, are shown for the PM and cytosolic fractions of the fluorescent proteins across the cell.” However, no dashed lines are visible in the PDF version I have access to.
5. In Figure 3 part A, it would be better to change “Sequences of AMPKalpha2…” to “Sequences of the C-terminal portions of AMPKalpha2…”.
6. On page 9 there is a typographical error: “The Afr6 GTPase”.

© 2022. Published by The Company of Biologists under the terms of the Creative Commons Attribution License (https://creativecommons.org/licenses/by/4.0/).

Reviewer 2

Advance summary and potential significance to field

This manuscript is a follow-up to a previous study from the authors' lab (Hsu et al, Nat Comm 2015), reporting the surprising finding that the yeast AMPK homolog Snf1 can act as a non-canonical guanine nucleotide exchange factor (GEF) for the GTPase Arf3. In that study, the authors reported that Snf1 activated Arf3 upon glucose deprivation, and promoted invasive growth in an Arf3-dependent manner.
Here they present evidence that mammalian (human) AMPK similarly activates the Arf3 homolog Arf6 and consequently stimulates the migration of prostate cancer (SKOV3) cells under energy-limiting (low glucose) conditions. AMPK lacks the canonical Sec7 homology domain found in other Arf GEFs including GEFs for Arf6, and mapping experiments suggest that, similar to Snf1, GEF activity is contained in the C-terminal regulatory domain of the AMPK alpha subunit and does not require its kinase activity.

Comments for the author

While the data are intriguing, there are several significant problems with the manuscript in its current form.
These include:
1. This study essentially confirms the results of the earlier one in the yeast system. While it is important to confirm that such mechanisms are evolutionarily conserved, the advance in our knowledge is incremental.
2. The GEF assays (Fig. 1) are suspect. First, the Arf is not myristoylated, which it would be in vivo.
Membranes are an important part of the activation mechanism; they displace the myristoylated amphipathic N-terminal helix, allowing access of the GEF to the Arf nucleotide binding pocket. It is not clear from the methods or figure legends whether the recombinant Arf6 used here contains its N-terminal helix or not. Related to this point, there are no membranes present in these assays. Membranes are typically supplied for GEF assays in the form of liposomes. The well-characterized Arf6 GEF ARNO, which is used here as a positive control, is actually autoinhibited in the absence of membranes, as shown by others. It is plausible that AMPK does in fact possess GEF activity, but this needs to be shown more rigorously.
3. Related to point 2, the cell-based GEF assays (Fig. 1C-F) are intriguing, and do suggest that AMPK has a role in Arf6 activation, but given the deficiencies in the in vitro GEF assays it is hard to say whether this is direct or not. The observation that Arf6 activation does not require AMPK catalytic activity is suggestive, but not definitive proof that AMPK directly activates Arf6.
4. The immunofluorescence data (Fig. 2B) are not convincing. Arf6 is largely on the plasma membrane to start with, and there is no obvious change in distribution upon glucose deprivation. Some translocation of AMPK is suggested by the image shown, but this is not quantified. The figure legend refers to linear profiles used for quantitation but none are shown. The cell fractionation data (Fig.2A) suggest a small increase in membrane association, but there also appears to be a mobility shift in Arf6 upon glucose starvation that is not mentioned in the text. Is this real? Also, is this endogenous Arf6 or transfected tagged Arf6?
5. Fig. 3C – the authors state that expression of AMPK mutants lacking the Arf6 binding site fail to restore activity in AMPK-depleted cells. While activity does appear to be attenuated somewhat, it is still significantly higher than non-expressing controls, suggesting that other areas of AMPK may also contribute to Arf6 activation. This figure also lacks a glucose + control.
6. The results of the transwell migration assay (Fig. 4A) are confusing. First, why does AMPK depletion enhance migration in glucose-+ conditions but inhibit it in glucose-deficient conditions? Second, depletion of ARNO, which acts on Arf6, severely inhibits migration in both glucose-+ and glucose-deficient conditions, while the effect of AMPK depletion is only partial and only observed in glucose-deprived cells.
The authors assume that the effects of AMPK knockdown are due to failure to activate Arf6, but do not show a corresponding Arf6 knockdown in their migration assays.
7. The defect in wound closure (Fig. 4C) is not dramatic, and needs to be quantified in any case. This may be due to sampling at a single time point (40h) after wounding. Sampling at multiple time points might reveal a more significant delay in wound closure that occurs early, but recovers at later stages.

© 2022. Published by The Company of Biologists under the terms of the Creative Commons Attribution License (https://creativecommons.org/licenses/by/4.0/).

Reviewer 3

Advance summary and potential significance to field

This is an interesting study which proposes that AMPK can function as a GEF for Arf6 during glucose starvation to promote cell invasion and migration. This work is of interest especially to the fields of cell invasion and metabolism. However several issues need to be addressed.

Comments for the author

1. All of this work was done using one cell line. Additional cell lines should be included for key findings (effect on Arf6 activation, invasion migration).
2. The authors demonstrate a synergistic reduction of Arf6 activation with knockdown of AMPKα1 and AMPKα2. Does this synergistic effect hold true in other cell types.
3. What is the effect knockdown of AMPKα1 and AMPKα2 or both, on Arf6 activation without glucose deprivation.
4. The authors should directly demonstrate that AICAR effect on Arf6 activation requires AMPK, for example by use of Arf6 binding defective AMPK mutants or knockdown.
5. The data presented in figure 2 is interesting. The colocalization of Arf6 and AMPK should be better supported by quantitation, for example by providing colocalization coefficients.
6. The AMPKa2-WT-mediated rescue of cell invasion is of interest and an important piece of data, but the same approach should be used to demonstrate rescue of Arf6 activation to justify the conclusions drawn. Further, this study in general would be enhanced by inclusion of more than a single shRNA sequence.
7. The authors conclude that AMPK acts upstream of Arf6 during cell migration and invasion. This would be better supported by demonstrating that knockdown or expression of dominant negative Arf6 blocks invasion with upregulation of AMPK.
8. What is the effect of AMPK activation on cell invasion upon glucose deprivation?
9. The authors should place their findings in the context of the field in the Results & Discussion section.

First revision

Author response to reviewers' comments

Reply to the Reviewers

We deeply thank all reviewers for their critical assessment of our work. Each also provided positive comments that suggest how we can further improve our study. We have carefully performed many additional experiments. These are described below as part of the point-by-point response to all comments of the last review.

Reviewer 1

1. *On page 8, first paragraph, the authors use the term "GGA3-PBD (1-316 a.a.)", without defining "PBD". Is it the same as the GGA3 portion of the fusion protein defined on page 9: "Golgi- associated gamma-adaptin ear-containing the ARF binding protein 3-GST (GGA3-GST) fusion protein". In the figure legends, the GGA3 pull-down experiments are indicated as being performed by "GST-GGA3", with "GST-GGA3-PBD" used in the figure panels. The same nomenclature should be used throughout, and defined clearly the first time the construct is described.*

   Thank you for pointing out this issue. We clearly defined the GST-GGA3-PBD construct with unified nomenclature. Briefly, we took advantage of the GGA3-PBD fragment containing the VHS- and ARF-binding domains of GGA3 (1-316 a.a.) to detect active ARF6 (Dell'Angelica et al. 2000, Li et al. 2007).

© 2022. Published by The Company of Biologists under the terms of the Creative Commons Attribution License (https://creativecommons.org/licenses/by/4.0/).

The primer pairs for amplification of GGA3-PBD are shown below.
GGA3-VHS-GAT5’ GAATTCATGGCGGAGGCGGAAGGG
GGA3-VHS-GAT3’ GTCGACGTCAGGCAGGGTTAAGGTAG

Dell'Angelica, E C et al. (2000)“GGAs: a family of ADP ribosylation factor-binding proteins related to adaptors and associated with the Golgi complex.” The Journal of cell biology 149: 81- 94.

Li CC, Chiang TC, Wu TS, Pacheco-Rodriguez G, Moss J, Lee FJ. (2007) ARL4D recruits cytohesin-2/ARNO to modulate actin remodeling. Mol Biol Cell. 18:4420-37.

2. *In Fig 1, why is there only one band in the Coomassie gel for GGA3-PBD in part C, but two bands in part E. I am assuming it is because the gels used were different, or the run times were different, but it would be good to explain this difference.*

   The difference in the GST-GGA3-PBD pattern between Fig 1C and 1E is due to the different cropping gel images shown below. We are now using the same cropping strategy for those gel images as shown in the Figure 1C and 1E.

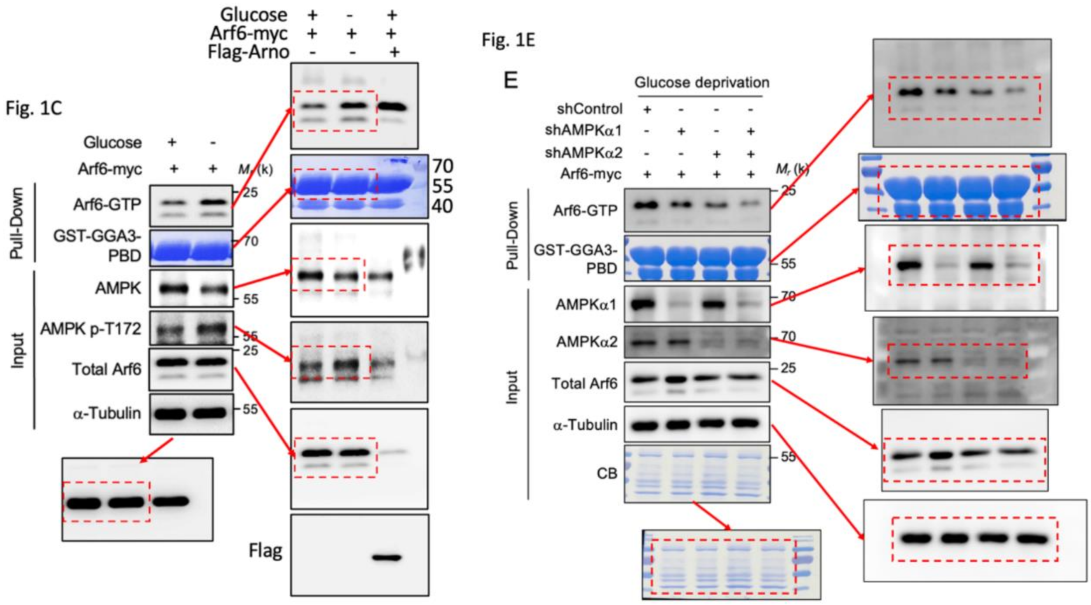

3. *At the top of page 6, the authors state “These data indicate that AMPK activates Arf6 in a kinase- independent manner and that phosphorylation on Thr172 of AMPK is required for this activation process.” Presumably the phosphorylation of AMPK Thr172 requires kinase activity, although it is clearly not an autophosphorylation. It would be better to say something like “AMPK activation of Arf6 does not require its kinase domain”.*

   We greatly appreciate the reviewer’s comment. We have rephrased the description as suggested by the reviewer to make our point clearer.

4. *Figure 2B. The authors state “Linear profiles, as indicated by the dashed lines created using ImageJ software, are shown for the PM and cytosolic fractions of the fluorescent proteins across the cell.” However, no dashed lines are visible in the PDF version I have access to.*

   We apologize for the missing linear profiles shown below. We have added these data to the edited Figure 2B.

5. *In Figure 3 part A, it would be better to change “Sequences of AMPKalpha2…” to “Sequences of the C-terminal portions of AMPKalpha2…”.*
   As suggested by the reviewer, we have changed the statement.

© 2022. Published by The Company of Biologists under the terms of the Creative Commons Attribution License (https://creativecommons.org/licenses/by/4.0/).

6. *On page 9 there is a typographical error: "The Afr6 GTPase".*

We have corrected the error.

Reviewer 2

1. *This study essentially confirms the results of the earlier one in the yeast system. While it is important to confirm that such mechanisms are evolutionarily conserved, the advance in our knowledge is incremental.*

Although we conclude that AMPK activates Arf6 in mammalian cells, which also confirmed the study in a yeast system, we wish to clarify that we have discovered more than the conserved machinery. In our current study, we further provide two significant results: (i) We have shown that treatment with the canonical AMPK activator AICAR, a nucleoside analog that elevates its kinase activity, also activates Arf6 (edited Figure 1F). Arf6 is typically activated by the conserved Sec7 domain-containing protein through its glutamic finger in the hydrophobic groove for nucleotide exchange (Macia EM, et al, 2001). Although AMPK does not appear to possess a Sec7 domain structurally, our study demonstrates a novel mechanism for nucleotide exchange of Arf small GTPase by the AMPK activator AICAR. (ii) We also demonstrated that Arf6 activation by AMPK is highly correlated with cancer cell migration and invasion (Figure 4), indicating the pathophysiological implication of AMPK and Arf6 in the disease model. We believe that these two cellular phenomena, driven by AMPK and Arf6, are of both importance and significance beyond the scope of evolutionary conservation between yeast and mammalian systems. We pointed out the significance of our findings in the conclusion section.

Macia, E., M. Chabre, and M. Franco. (2001) Specificities for the small G proteins ARF1 and ARF6 of the guanine nucleotide exchange factors ARNO and EFA6. J Biol Chem. 276:24925-24930.

2. *The GEF assays (Fig. 1) are suspect. First, the Arf is not myristoylated, which it would be in vivo. Membranes are an important part of the activation mechanism; they displace the myristoylated, amphipathic N-terminal helix, allowing access of the GEF to the Arf nucleotide binding pocket. It is not clear from the methods or figure legends whether the recombinant Arf6 used here contains its N-terminal helix or not. Related to this point, there are no membranes present in these assays. Membranes are typically supplied for GEF assays in the form of liposomes. The well-characterized Arf6 GEF ARNO, which is used here as a positive control, is actually autoinhibited in the absence of membranes, as shown by others. It is plausible that AMPK does in fact possess GEF activity, but this needs to be shown more rigorously.*

This comment highlights the membrane supply for the GEF assay. With the respect to the results being mentioned, we first thank the reviewer for pointing out the lack of description of the recombinant proteins used in the GEF assay. In Figure 1A and 1B, we used Arf6-dN13 forms, which bypass the requirement of myristoylation and become more flexible to access the GEF without membrane incubation, as the substrate to perform the GEF assay. In this case, previous reports have also provided evidence that GEF could function well *in vitro* in the absence of membranes or liposomes (Beraud-Dufour, Robineau et al. 1998, Bar-Peled, Schweitzer et al. 2012). For the production of His-Arf6-dN13 protein, the construct was transformed into BL21 cells. The induction of recombinant His-Arf6-dN13 was initiated by the addition of 0.5 mM isopropyl β-D- 1-thiogalactopyranoside (IPTG) followed by incubation for 3 h at 37°C. The cell pellets were then suspended with lysis buffer containing lysozyme and incubated on ice for 30 min, lysed with a nitrogen bomb at a pressure of 1500 pounds per square inch, and then the debris was removed by centrifugation (14,000 x g, 30 min). The resulting supernatant was bound with Ni^{2+}-NTA agarose (Qiagen, 1018244) at 4°C for 2 h, and His-tagged proteins were eluted in buffer containing 100 mM imidazole. Fractions were collected and analyzed by SDS-PAGE and Coomassie Blue staining to identify and evaluate target protein purity and concentration. Nevertheless, we very much appreciate the reviewer's suggestion and have described more details about the protein preparation for the GEF assay.
Bar-Peled, L., L. D. Schweitzer, R. Zoncu and D. M. Sabatini (2012). "Ragulator is a GEF for the rag GTPases that signal amino acid levels to mTORC1." Cell 150: 1196-1208.

© 2022. Published by The Company of Biologists under the terms of the Creative Commons Attribution License (https://creativecommons.org/licenses/by/4.0/).

Beraud-Dufour, S., S. Robineau, P. Chardin, S. Paris, M. Chabre, J. Cherfils and B. Antonny (1998). "A glutamic finger in the guanine nucleotide exchange factor ARNO displaces Mg2+ and the beta-phosphate to destabilize GDP on ARF1." EMBO J 17: 3651-3659.

3. *Related to point 2, the cell-based GEF assays (Fig. 1C-F) are intriguing, and do suggest that AMPK has a role in Arf6 activation, but given the deficiencies in the in vitro GEF assays it is hard to say whether this is direct or not. The observation that Arf6 activation does not require AMPK catalytic activity is suggestive, but not definitive proof that AMPK directly activates Arf6.*

   For the interaction analysis, we first showed that AMPK directly interacts with inactive Arf6 (both Arf6-GDP and Arf6-d13N-T27N) both *in vitro* and *in vivo* (Figure S1). We further showed that the C-terminal regulatory domain of AMPK could only directly activate Arf6 *in vitro* (Figure 1A and 1B), suggesting that AMPK could be a direct nucleotide exchange factor to activate Arf6. Furthermore, the catalytically dead mutant AMPK-K45R also possesses GEF activity for Arf6 activation *in vivo* (Figure 2D). Collectively, these data demonstrate that AMPK truly activates Arf6 directly independent of its kinase activity.

4. *The immunofluorescence data (Fig. 2B) are not convincing. Arf6 is largely on the plasma membrane to start with, and there is no obvious change in distribution upon glucose deprivation. Some translocation of AMPK is suggested by the image shown, but this is not quantified. The figure legend refers to linear profiles used for quantitation but none are shown. The cell fractionation data (Fig.2A) suggest a small increase in membrane association, but there also appears to be a mobility shift in Arf6 upon glucose starvation that is not mentioned in the text. Is this real? Also, is this endogenous Arf6 or transfected, tagged Arf6?*

   First, we apologize for the issue of missing linear profiles and we have added those data in the revised version (edited Figure 2B). Fluorescence intensity may vary due to the expression level of a protein and/or the antibody used in each experiment. Such variable possibilities may further lead to misunderstanding of the change in protein distribution when primary images are simply viewed by eyes. To further provide an objective evaluation of Arf6 localization, we further performed a quantification test for the protein colocalization (edited Figure 2C). Regarding the band shift of Arf6, we realize that the mobility shift is due to the overall gel mobility for that sample. We have replaced this image with another gel image to avoid confusion (edited Figure 2A).

5. *Fig. 3C – the authors state that expression of AMPK mutants lacking the Arf6 binding site fail to restore activity in AMPK-depleted cells. While activity does appear to be attenuated somewhat, it is still significantly higher than non-expressing controls, suggesting that other areas of AMPK may also contribute to Arf6 activation. This figure also lacks a glucose + control.*

   Thank you for the comments. In Fig. 3C, the level of active Arf6 in AMPK-A2- and AMPK-A5-expressing cells appears to be higher than that in siAMPKa2-treated cells. We reason that AMPK-A2- and AMPK-A5-expressing cells have higher levels of exogenous Arf6 compared to siAMPKa2-treated cells. However, we cannot rule out that other regions of AMPK may also contribute to Arf6 activation. For the glucose+ control, we have shown this comparison in Figures 1C, 1D, new S2A, and new S2B and the major purpose of Figure 3C is to examine the active Arf6 level in cells expressing AMPK-WT, A2, and A5.

6. *The results of the transwell migration assay (Fig. 4A) are confusing. First, why does AMPK depletion enhance migration in glucose-+ conditions but inhibit it in glucose-deficient conditions? Second, depletion of ARNO, which acts on Arf6, severely inhibits migration in both glucose-+ and glucose-deficient conditions, while the effect of AMPK depletion is only partial and only observed in glucose-deprived cells. The authors assume that the effects of AMPK knockdown are due to failure to activate Arf6, but do not show a corresponding Arf6 knockdown in their migration assays.*

   We appreciate the reviewer's comment on the role of AMPK in cell migration and invasion. We would like to point out that cell migration and invasion are complicated, as evidenced by

© 2022. Published by The Company of Biologists under the terms of the Creative Commons Attribution License (https://creativecommons.org/licenses/by/4.0/).

discrepancies in AMPK-mediated cell migration and invasion results from several studies. Specifically, glucose starvation has been shown to induce cell migration and invasiveness through AMPK activity (Endo, Owada et al. 2018). We also found that AMPK is critical in regulating cell migration and invasion (Figure 4A-C), suggesting that AMPK functions as a GEF for Arf6 activation under glucose starvation to promote cell invasion and migration. We already provided additional Arf6 knockdown as the control group in the migration assay (Sup. Figure S3A).

Endo, H., S. Owada, Y. Inagaki, Y. Shida and M. Tatemichi (2018). "Glucose starvation induces LKB1-AMPK-mediated MMP-9 expression in cancer cells." Sci Rep 8: 10122.

7. *The defect in wound closure (Fig. 4C) is not dramatic, and needs to be quantified in any case. This may be due to sampling at a single time point (40h) after wounding. Sampling at multiple time points might reveal a more significant delay in wound closure that occurs early, but recovers at later stages.*

As the reviewer requested, we have examined earlier time points to examine the wound closure and found that none of the different time points were that dramatic for cell migration (Figure S3C and 4C). AMPK-mediated cell migration involves multiple pathways driven by the combination of various substrates. How these signaling pathways coordinate to contribute to cell migration still needs to be further investigated.

Reviewer 3

1. *All of this work was done using one cell line. Additional cell lines should be included for key findings (effect on Arf6 activation, invasion, migration).*

As suggested by the reviewer, we examined the effect of AMPK on Arf6 activation in 293T cells and found that both expression of AMPK and treatment with the AMPK activator AICAR significantly enhanced Arf6 activity in 293T cells (new Figure S2B-D). We further used 293T cells to observe the effect of glucose starvation in cell migration and invasion assays. However, we found that 293T cells are not compatible with the cell motility assay, because 293T cells are well- known for attaching poorly and have a tendency to detach from the plate.

We also used HeLa cells to test the effect of glucose starvation and found that glucose starvation seems not to be responsible for cell migration and invasion in AMPK knockdown HeLa cells (as shown below). We speculate that, upon glucose starvation, the AMPK-Arf6 axis may not be that dominant in controlling cell invasion and migration in HeLa cells. We, therefore, used SKOV3 cells for cell invasion and migration assays.

© 2022. Published by The Company of Biologists under the terms of the Creative Commons Attribution License (https://creativecommons.org/licenses/by/4.0/).

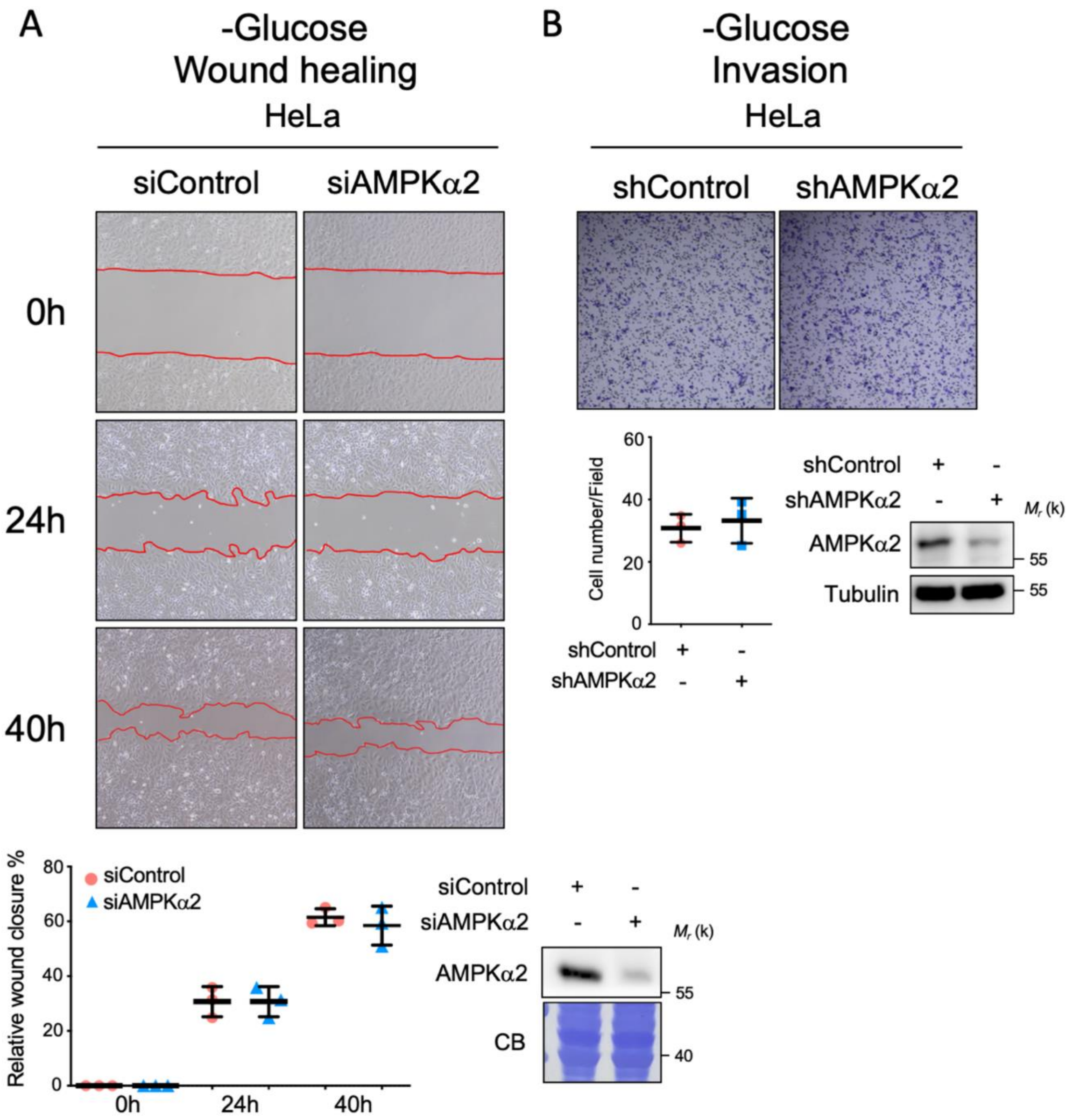

2. *The authors demonstrate a synergistic reduction of Arf6 activation with knockdown of AMPKa1 and AMPKa2. Does this synergistic effect hold true in other cell types.*

   As the reviewer requested, we further used 293T cells to observe the synergistic effect and also found a synergistic reduction of Arf6 activity in 293T cells with the co-knockdown of AMPKα1 and AMPKα2 (new Figure S2B).

3. *What is the effect knockdown of AMPKa1 and AMPKa2 or both, on Arf6 activation without glucose deprivation.*

   As the reviewer suggested, we detected less active Arf6 in siAMPKα1- and AMPKα2-treated cells under normal glucose conditions (Figure S2A and S2B). Our data also showed that knockdown of both AMPKα1 and α2 upon glucose starvation resulted in a synergistic reduction of active Arf6 (Figure 1E). These data suggest that there is basal GEF activity of AMPK under normal glucose conditions, and that glucose starvation significantly induces AMPK GEF activity for Arf6 activation.

4. *The authors should directly demonstrate that AICAR effect on Arf6 activation requires AMPK, for example by use of Arf6 binding defective AMPK mutants or knockdown.*

© 2022. Published by The Company of Biologists under the terms of the Creative Commons Attribution License (https://creativecommons.org/licenses/by/4.0/).

As suggested by the reviewer, we detected a significant reduction in active Arf6 levels in siAMPK-cells treated with AICAR (edited Figure 1F and new S2C).

5. *The data presented in figure 2 is interesting. The colocalization of Arf6 and AMPK should be better supported by quantitation, for example by providing colocalization coefficients.*

   As suggested by the reviewer, we performed colocalization analysis for AMPK and Arf6 and demonstrated higher AMPK colocalization with Arf6 under glucose starvation (new Figure 2C).

6. *The AMPKα2-WT-mediated rescue of cell invasion is of interest and an important piece of data, but the same approach should be used to demonstrate rescue of Arf6 activation to justify the conclusions drawn. Further, this study in general would be enhanced by inclusion of more than a single shRNA sequence.*

   To address the reviewer's request, we also found that cell invasion was significantly inhibited by AMPK-depletion using a second independent siRNA against AMPKα2 (siAMPK¤2 #2) under glucose starvation (new Figure S3B). We also demonstrated that wild-type but not the A2 and A5 mutant forms of AMPK could rescue the active Arf6 level in AMPK knockdown cells (Figure 3C). Collectively, these data suggest that AMPK binding to Arf6 for its activation is required for cell invasion under glucose starvation.

7. *The authors conclude that AMPK acts upstream of Arf6 during cell migration and invasion. This would be better supported by demonstrating that knockdown or expression of dominant negative Arf6 blocks invasion with upregulation of AMPK.*

   As suggested by the reviewer, we added data in Figure S3A to show that knockdown of Arf6 in cells with AMPK activation upon glucose starvation decreased cell invasion.

8. *What is the effect of AMPK activation on cell invasion upon glucose deprivation?*

   AMPK activity is considered as the master regulator that controls cellular events upon glucose starvation or energy stress. A previous study has demonstrated that AMPK activity is critical in the regulation of cell migration and invasion in breast and lung cancer cells (Saxena, Balaji et al. 2018). Furthermore, glucose starvation has been shown to induce cell migration and invasiveness through AMPK-mediated MMP-9 expression (Endo, Owada et al. 2018). AMPK is also required for microtubule dynamics to control directional cell migration (Nakano, Kato et al. 2010). These studies all suggest that AMPK activation facilitates its downstream signaling and/or gene expression to account for cell migration and invasion. In our current study, we further manifest that AMPK, in addition to AMPK kinase activity, can function as a non-kinase role to promote Arf6 activity for the induction of cell migration and invasion. We added this discussion in the revised text.

Endo, H., S. Owada, Y. Inagaki, Y. Shida and M. Tatemichi (2018). "Glucose starvation induces LKB1-AMPK-mediated MMP-9 expression in cancer cells." Sci Rep 8: 10122.
Nakano, A., H. Kato, T. Watanabe, K. D. Min, S. Yamazaki, Y. Asano, O. Seguchi, S. Higo, Y. Shintani, H. Asanuma, M. Asakura, T. Minamino, K. Kaibuchi, N. Mochizuki, M. Kitakaze and S. Takashima (2010). "AMPK controls the speed of microtubule polymerization and directional cell migration through CLIP-170 phosphorylation." Nat Cell Biol 12: 583-590.
Saxena, M., S. A. Balaji, N. Deshpande, S. Ranganathan, D. M. Pillai, S. K. Hindupur and A. Rangarajan (2018). "AMP-activated protein kinase promotes epithelial-mesenchymal transition in cancer cells through Twist1 upregulation." J Cell Sci 131(14):jcs208314

9. *The authors should place their findings in the context of the field in the Results & Discussion section.*

   As suggested by the reviewer, we have added more discussion to combine our findings with previous studies regarding AMPK activation and Arf6 activity in the Results & Discussion section indicated in red.

© 2022. Published by The Company of Biologists under the terms of the Creative Commons Attribution License (https://creativecommons.org/licenses/by/4.0/).

Second decision letter

MS ID#: JOCES/2021/259609

MS TITLE: AMPK promotes Arf6 activation in a kinase-independent manner upon energy deprivation

AUTHORS: Kuan Jung S Chen, Jia-Wei Hsu, and Fang-Jen S Lee
ARTICLE TYPE: Short Report

We have now reached a decision on the above manuscript.

To see the reviewers' reports and a copy of this decision letter, please go to: https://submit-jcs.biologists.organd click on the 'Manuscripts with Decisions' queue in the Author Area. (Corresponding author only has access to reviews.)

As you will see, the reviewers are more positive but they raised some points that I feel are important to address. In particular, I think the paper would be greatly strengthened by GEF assays in the presence of liposomes (reviewer 2). Reviewer 3 requests additional experiments addressing differences you observe between cell types. I feel that pinning down the reason for such differences might not be easy, so in my mind these experiments are not required for publication. I hope that you will be able to carry out a few GEF assays with lipids because I would like to be able to accept your paper.

Please ensure that you clearly highlight all changes made in the revised manuscript. Please avoid using 'Tracked changes' in Word files as these are lost in PDF conversion.

I should be grateful if you would also provide a point-by-point response detailing how you have dealt with the points raised by the reviewers in the 'Response to Reviewers' box. Please attend to all of the reviewers' comments. If you do not agree with any of their criticisms or suggestions please explain clearly why this is so.

Reviewer 1

Advance summary and potential significance to field

This manuscript is an excellent, interesting and important study that reports a new guanine nucleotide exchange factor (activator) for the Arf family of small G proteins that is implicated in cancer cell migration and invasion. To date, only one major family of exchange factors for Arf family proteins have been identified, the Sec7 domain proteins. AMPK, which is a key regulator of cellular metabolism, is identified in this study as an exchange factor for mammalian Arf6, which is important for Arf6 capacity to promote cell migration and invasion. This group previously identified the yeast orthologue of AMPK as a GEF for Arf proteins in yeast, and here show that AMPK is an evolutionarily conserved activator of Arf proteins, which activates Arf6 in mammalian cells upon energy deprivation. Arf6 is an important regulator of membrane trafficking and cytoskeleton dynamics at the plasma membrane, and promotes cancer cell migration and invasiveness. In this study, the authors very clearly and convincingly demonstrate that the C-terminal region of the catalytic subunit of AMPK stimulates nucleotide exchange on Arf6 both in vitro and in vivo. In addition, the authors show that Arf6 activation by AMPK promotes cancer cell migration and invasion, a novel result that has important implications for understanding cancer disease mechanisms in humans.

Comments for the author

The authors have carried out a number of additional experiments, and modified the text of their manuscript, addressing all of my comments and those of the other reviewers. This important and comprehensive study is now fully acceptable for publication.

© 2022. Published by The Company of Biologists under the terms of the Creative Commons Attribution License (https://creativecommons.org/licenses/by/4.0/).

Reviewer 2

Advance summary and potential significance to field

This revised manuscript presents data suggesting that the energy-sensing kinase AMPK contributes to cell motility by activating the small GTPase ARF6. If correct, this would be the first evidence of an ARF6 GEF lacking the canonical Sec7 homologous catalytic domain found in all other ARF GEFs. Unfortunately the data supporting the GEF activity of AMPK is insufficient to draw this conclusion. As noted in my review, such assays need to use full length myristoylated ARF in the presence of acidic lipids to draw any convincing conclusion. This is the standard in the field, and although this concern was raised in the original review the authors did not address it here.

While the combination of biochemical and functional (migration) assays suggest that AMPK may indeed contribute to cell migration, its impact appears to be minor, and only detectable in only some cell types.

Comments for the author

This revised manuscript is improved over the original version but some of the most important aspects of the original concerns remain unresolved. The most important of these relates to the in vitro GEF assays (Fig. 1). As noted in my review, the standard in the field is to use myristoylated ARF and to include liposomes containing acidic lipids. All nucleotide exchange on ARFs in vivo takes place at membrane surfaces, and the lipid is an important component of the reaction. This is especially important for ARF6 which is membrane bound in both the GTP- and GDP-bound states. I mentioned in my original review that ARNO, which is used here as a positive control, is autoinhibited in the absence of negatively charged lipids. Although some ARNO activity is detectable in Fig. 1 it is likely to be orders of magnitude less than it would be in the presence of lipids. The data shown in Fig. 1 imply that the nucleotide exchange activity of AMPK is equivalent to that of ARNO, but it is very unlikely that this represents reality. These assays MUST be done in the presence of acidic lipids (typically PC spiked with 3-5% PIP2) using myristoylated ARF6 to have any relevance. The ΔdN13 mutant used by the authors (which lacks the entire N-terminal helix) is not an acceptable substitute. This gets at the issue of whether the ability of AMPK to activate ARF6 is direct or indirect. It remains possible that the C-terminus of AMPK simply interacts with a known ARF6 GEF and enhances its activity.
The immunofluorescence images and quantitation (Fig. 2B, C) are still not convincing. Lines could be drawn through any of the images shown that would show the opposite result. The quantitation shown in Fig. 2C is not convincing. Both proteins are widely distributed throughout the cell (as measured by the high colocalization in the absence of glucose). The small increase in colocalization observed after glucose addition could easily be due to biased selection of cells for quantitation. The shift in membrane-bound ARF6 in Fig. 2A is also very small, although amplified by the scale used for quantitation. As noted above ARF6 is membrane bound in both the GDP- and GTP-bound states, so it is not clear what this small increase in membrane association is meant to demonstrate. Finally, the authors refer to the AMPK-mediated activation of ARF6 as “critical” for cell migration, when in fact the inhibition observed in the absence of AMPK is quite small. While AMPK may in fact contribute to migration, its contribution seems small compared to other factors.

Reviewer 3

Advance summary and potential significance to field

The study suggests that AMPK can function as a GEF for the nucleotide exchange on the small GTP binding protein, Arf6, during glucose starvation to promote cell invasion and migration. This work is of interest especially to the fields of cell invasion and metabolism.

Comments for the author

The authors have addressed the concerns raised in my previous review for the most part. However it is very important that they address the differences seen (on Arf6 activation, invasion, migration) between the cell lines (Hela, relative to 293T and SKOVs) in the manuscript. Context matters.

© 2022. Published by The Company of Biologists under the terms of the Creative Commons Attribution License (https://creativecommons.org/licenses/by/4.0/).

Given the differences, I suggest the addition of additional cell lines. Presently the data with 293T and SKOV cell lines are described as a universal generalization.

Second revision

Author response to reviewers' comments

Reply to Reviewers
Reply to Reviewer #2

We sincerely appreciate the reviewer's constructive comments and have attempted to address each of the specific points as described below:

Reviewer 2 Comments for the author
This revised manuscript is improved over the original version but some of the most important aspects of the original concerns remain unresolved. The most important of these relates to the in vitro GEF assays (Fig. 1). As noted in my review, the standard in the field is to use myristoylated ARF and to include liposomes containing acidic lipids. All nucleotide exchange on ARFs in vivo takes place at membrane surfaces, and the lipid is an important component of the reaction. This is especially important for ARF6, which is membrane bound in both the GTP- and GDP-bound states. I mentioned in my original review that ARNO, which is used here as a positive control, is autoinhibited in the absence of negatively charged lipids. Although some ARNO activity is detectable in Fig. 1 it is likely to be orders of magnitude less than it would be in the presence of lipids. The data shown in Fig. 1 imply that the nucleotide exchange activity of AMPK is equivalent to that of ARNO, but it is very unlikely that this represents reality. These assays MUST be done in the presence of acidic lipids (typically PC spiked with 3-5% PIP2) using myristoylated ARF6 to have any relevance. The ΔdN13 mutant used by the authors (which lacks the entire N-terminal helix) is not an acceptable substitute. This gets at the issue of whether the ability of AMPK to activate ARF6 is direct or indirect. It remains possible that the C-terminus of AMPK simply interacts with a known ARF6 GEF and enhances its activity.

Response:
We appreciate the reviewer's comment on the direct role of GEF activity for Arf6 activation with membrane supply. We would like to clarify that the in vitro GEF activity assay has been done using purified recombinant Arf6-dN13, ARNO, and AMPK-C from E. coli. The biochemistry presented in our manuscript is quite clean and convincing for specific GEF interactions/activities between all of the proteins assayed. Nevertheless, considering the requirement of the membrane environment for GEF activity, we first purified myristoylated Arf6 and generated liposomes containing 45% PC, 20% PE, 30% PS, and 5% PIP2 (Chavrier and Franco, 2001; Stalder et al., 2011). We then used a fluorescence method to measure the dissociation of mant-GDP and the association of mant-GppNp on Myr-Arf6. All reactions were carried out at 50 mM HEPES (pH 7.5), 1 mM MgCl2, 2 mM EDTA, 100 mM KCl, and 1 mM DTT, using an excitation wavelength of 360 nm and emission at 440 nm. Our results demonstrate that C-terminal AMPK, comparable to the effect of ARNO, directly activates Arf6 in vitro (new Figure 1A and 1B).

Reference:
Chavrier, P. and Franco, M. (2001). Expression, purification, and biochemical properties of EFA6, a Sec7 domain-containing guanine exchange factor for ADP-ribosylation factor 6 (ARF6). Methods Enzymol 329, 272-9.

Stalder, D., Barelli, H., Gautier, R., Macia, E., Jackson, C. L. and Antonny, B. (2011). Kinetic studies of the Arf activator Arno on model membranes in the presence of Arf effectors suggest control by a positive feedback loop. J Biol Chem 286, 3873-83.

The immunofluorescence images and quantitation (Fig. 2B, C) are still not convincing. Lines could be drawn through any of the images shown that would show the opposite result. The quantitation shown in Fig. 2C is not convincing. Both proteins are widely distributed throughout the cell (as measured by the high colocalization in the absence of glucose). The small increase in colocalization

© 2022. Published by The Company of Biologists under the terms of the Creative Commons Attribution License (https://creativecommons.org/licenses/by/4.0/).

observed after glucose addition could easily be due to biased selection of cells for quantitation. The shift in membrane-bound ARF6 in Fig. 2A is also very small, although amplified by the scale used for quantitation. As noted above, ARF6 is membrane bound in both the GDP- and GTP-bound states, so it is not clear what this small increase in membrane association is meant to demonstrate.

Response:
We appreciate the reviewer's constructive comments. With respect to the specific images and quantitation being questioned (Figure 2B and 2C), we first wish to point out that our quantitation is compiled through software analysis and the distribution of the indicated proteins show very similar pattern with multiple experiments. Nevertheless, as further reassurance, we have re-performed the quantitation with more cells and have generated similar results in new Figure 2C. As demonstration, we show representative images to confirm the membrane enrichment of Arf6 upon glucose starvation. Our active Arf6 pull-down assay also indicated that glucose starvation increases active Arf6 in cells. Taken together, both immunofluorescence images and biochemical assays strongly suggest that more Arf6 is activated and translocated onto the plasma membrane upon glucose starvation.

Finally, the authors refer to the AMPK-mediated activation of ARF6 as "critical" for cell migration, when in fact the inhibition observed in the absence of AMPK is quite small. While AMPK may in fact contribute to migration, its contribution seems small compared to other factors.

Response:
We appreciate the reviewer's comments. We would like to highlight that Arf6 activation by AMPK is critically important to promote cancer cell migration specifically under "glucose deprivation" conditions (Figure 4A). AMPK may not be the only key factor to affect cell migration and invasion; however, the AMPK-Arf6 axis definitely contributes to cell invasion as we found expression of Arf6-binding detective mutants of AMPK, AMPK-A2, and AMPK2-A5, failed to rescue the migration and invasion abilities (Figure 4B, 4C S3C, and S3C). These data suggest that the migration and invasion ability in different types of cancer cells could be reflected by the AMPK-Arf6 axis upon glucose starvation.

Reply to Reviewer #3

We sincerely appreciate the reviewer's comments and have tried to address each of the specific points as described below:

Reviewer 3 Comments for the author
The authors have addressed the concerns raised in my previous review for the most part. However, it is very important that they address the differences seen (on Arf6 activation, invasion, migration) between the cell lines (Hela, relative to 293T and SKOVs) in the manuscript. Context matters. Given the differences, I suggest the addition of additional cell lines. Presently the data with 293T and SKOV cell lines are described as a universal generalization.

As the reviewer's suggestion, we have provided an additional cell line, OVCAR3, to demonstrate the AMPK-mediated Arf6 activation is important to regulate cell migration (new Fig S2A and S3C).

Third decision letter

MS ID#: JOCES/2021/259609

MS TITLE: AMPK promotes Arf6 activation in a kinase-independent manner upon energy deprivation

AUTHORS: Kuan Jung S Chen, Jia-Wei Hsu, and Fang-Jen S Lee
ARTICLE TYPE: Short Report

I am happy to tell you that your manuscript has been accepted for publication in Journal of Cell Science, pending standard ethics checks.

© 2022. Published by The Company of Biologists under the terms of the Creative Commons Attribution License (https://creativecommons.org/licenses/by/4.0/).
